# Understanding the experience, treatment preferences and goals of people living with chronic lymphocytic leukemia (CLL) in Australia

**DOI:** 10.1186/s12885-024-12589-9

**Published:** 2024-07-11

**Authors:** Simon Fifer, Jenni Godsell, Stephen Opat, Nada Hamad, Masa Lasica, Cecily Forsyth, Louisa Morand, Erica Smeaton, Sharon Winton, Andrea Puig, Marija McGeachie

**Affiliations:** 1 Community and Patient Preference Research (CaPPRe), Level 20, 50 Bligh Street, Sydney, NSW 2000 Australia; 2 Monash Health, 246 Clayton Road, Clayton, VIC 3168 Australia; 3 Department of Haematology, St Vincent’s Hospital Sydney, 390 Victoria Street, Darlinghurst, NSW 2010 Australia; 4 School of Clinical Medicine, Faculty of Medicine and Health, UNSW Sydney, High St, Kensington, NSW 2052 Australia; 5 School of Medicine, University of Notre Dame Sydney, 160 Oxford St, Darlinghurst, NSW 2010 Australia; 6 St Vincent’s Hospital Melbourne, 41 Victoria Parade, Fitzroy, VIC 3065 Australia; 7 Central Coast Haematology, 14 - 18 Jarrett Street, Gosford, NSW 2250 Australia; 8 Lymphoma Australia, PO Box 676, Fortitude Valley, QLD 4006 Australia; 9 Johnson and Johnson, 66 Waterloo Road, Macquarie Park, NSW 2113 Australia

**Keywords:** Lymphoma, Discrete choice experiment, Shared decision making, Patient satisfaction, Best-worst scaling, Patient-centric care

## Abstract

**Background:**

Listening to patient voices is critical, in terms of how people experience their condition as well as their treatment preferences. This research explored the patient journey, therapy attributes and goals among treatment experienced adults with chronic lymphocytic leukemia (CLL). We sought to understand patient experiences, needs and expectations to identify areas for improvement of treatment and care delivery.

**Methods:**

Two online surveys were developed for completion by CLL patients. In Stage 1, participants completed a best-worst scaling (BWS) task to evaluate eleven previously validated healthcare journey moments that matter (MTM). Responses were used to generate the patient experience index (PEI) score. In Stage 2, participants completed a survey that included both a discrete choice experiment (DCE) to assess drivers of treatment preferences by evaluating the relative attribute importance (RAI) of seven features and a BWS exercise which explored long-term treatment goals.

**Results:**

Twenty-five patients completed Stage 1 and thirty patients Stage 2. Treatment experience was balanced between oral and intravenous medication. The most important/least satisfied MTM were treatment effectiveness, access to support and other treatments as well as monitoring progress. The median PEI score was 66.2 (out of 100). DCE results demonstrated that patients most value treatments for CLL that are associated with prolonged progression free survival (PFS; RAI: 24.6%), followed by treatments that have a lower risk of severe side effects and lower out-of-pocket costs (RAI: 19.5%, 17.4%, respectively). The remainder of the weight in decision making (38.5%) was split between the remaining attributes, namely ‘mild to moderate side effects’ (13.4%), ‘long-term risks’ (12.2%), type of treatment (i.e., oral, IV or a combination of oral and IV; 8.7%) and treatment duration (i.e., ongoing versus fixed; 4.2%). Patients preferred oral to intravenous therapy. The most valued long-term treatment goal was to be physically healthy, followed by living a long life, spending time with family/friends, and avoiding hospitalization.

**Conclusion:**

Treatment experienced patients with CLL are focused on receiving effective, safe therapies and value long PFS. Consideration and discussion of other attributes, such as once daily dosing, oral only medication, out-of-pocket costs and access to support services may affect patient treatment choices and ultimately enhance their healthcare experience and outcomes.

**Supplementary Information:**

The online version contains supplementary material available at 10.1186/s12885-024-12589-9.

## Background

Chronic lymphocytic leukemia (CLL) is a chronic lymphoproliferative disorder, classified as a subtype of lymphoma by the World Health Organization [1]. CLL is the most common adult blood cancer, with around 2,000 Australians diagnosed each year [2]. It is more common in males than in females and has an average age at diagnosis of 70 years [3]. The natural history of CLL varies, and many patients live with the disease for years before it progresses and treatment is required [3]. Several targeted therapy treatments and combinations have become available leading to chemotherapy-free regimens now being preferred for most patients with CLL, particularly those with high risk features [4, 5]. These agents include the Bruton’s tyrosine kinase inhibitors (BTKi’s: ibrutinib, acalabrutinib and zanubrutinib), BCL-2 inhibitor (venetoclax), and PI3K inhibitors (idelalisib, duvelisib) [5, 6]. The use of chemoimmunotherapy combinations such as fludarabine, cyclophosphamide and rituximab are declining [7].

To assess the differences between current CLL treatments, clinicians and patients will consider efficacy (including progression free survival (PFS) and overall survival (OS)), and side effect profile as well as other treatment attributes [8–10]. For example, oral therapies can improve access for patients in rural and regional areas since injectable therapy generally requires clinic attendance. Other attributes considered include frequency of dosing, duration of therapy (ongoing versus fixed), and out-of-pocket costs (for example private health insurance premiums, medication co-payments, carer support and the costs associated with travel and hospital visits). Access to support services for patients and their carer can also vary. These factors form part of each patient’s unique experience. With several novel therapies entering the Australian market in the last five years, it is important to understand how patients living with CLL feel about differences in treatment attributes, as they navigate a complex healthcare environment including chemotherapy-free regimens and chemoimmunotherapy combinations. Furthermore, it may be beneficial for clinicians to more broadly understand the potential barriers to care.

This research aimed to explore the patient journey, treatment preferences and long-term treatment goals among patients who have received treatment for CLL. A primary objective of this study was to develop a greater understanding of the patient experience along the healthcare pathway (including experiences relating to diagnosis, treatment and monitoring, involvement in decision making, the quality of the healthcare team, provision of holistic care, support for carers and out-of-pocket costs). Further objectives were to understand treatment preferences and treatment goals among this patient group. The research sought to uncover what is important to patients along the healthcare pathway and how satisfied patients are with different areas of treatment and care. The study also focused on the relative importance of therapy attributes in order to better characterize treatment decision making at a patient level. The final objective of this study was to explore what CLL patients value most as long-term treatment outcomes and how these relate to treatment decision making.

## Methods

### Design

#### Development of survey instruments

A discrete choice experiment (DCE) [11, 12] and best-worst scaling (BWS) exercises [13] were developed for use in this study. DCEs are used to model the trade-offs and preferences revealed by the choices that people make. DCEs utilize a survey (which CaPPRe administers via an online questionnaire) that presents participants with hypothetical treatment scenarios which may or may not map to actual treatment options in the market. In each scenario participants are asked to choose an alternative within each scenario that maximizes their utility (i.e., satisfaction), according to their own value framework. BWS tasks avoid many of the scaling problems associated with Likert ratings (where the participant rates a statement on a scale of 5 or 7 responses). In BWS exercises participants are shown different combinations of statements and asked to select the best and worst option in each comparison. Analyzing the observed choices using statistical models allows researchers to quantify the hierarchy and prioritization of statements on a common scale.

To identify the most important attributes, levels and treatment goals for inclusion in the quantitative survey, DCE and BWS statements, a literature review was undertaken that explored previous preference studies to guide the development of attributes and levels appropriate for inclusion in this specific study. Furthermore, clinical reviews of Phase 3 studies of sufficient relevance and quality were reviewed to ensure proposed attributes were in line with relevant clinical endpoints of CLL research. Additionally, qualitative interviews with six patients with CLL were initially undertaken to explore first-hand patient feedback on both the impact of living with CLL and the attributes and levels for inclusion in the quantitative instrument. Finally, a workshop provided expert opinion from three hematologists, and consultation with Lymphoma Australia, a health care organization that provides education and advocacy for CLL and lymphoma patients, ensured that the survey instrument was relevant and appropriately worded. The patient experience component of the survey instrument had been previously validated by CaPPRe through in-depth interviews and stakeholder workshops in several other therapeutic areas and was adapted to ensure relevance in CLL. Survey instruments have been included as Supplementary Materials 2 and 3. This research approach including the experimental design followed guidance provided by The International Society for Pharmacoeconomics and Outcomes Research (ISPOR) Good Research Practices for Conjoint Analysis Task Force [14, 15] and the United States Food and Drug Administration (FDA) [16, 17]. This study was approved by the Human Research Ethics Committee, Bellberry Limited on 23rd June 2022, Application number: 2022-05-448.

### Participants

Participants were adults living with CLL in Australia, who were compensated for their time. Participants were recruited through a mixture of channels including a patient support organization (Lymphoma Australia), a health social network (Health Unlocked), a pharmaceutical company patient support program (Johnson and Johnson), two specialist research recruitment organizations (CRNRSTONE and I-Link Research) and through private clinician referrals. Information on the participants’ diagnosis was self-reported and collected through the initial screening questionnaire. The quantitative research was conducted in two stages, with eligible participants being able to take part in one or both stages. While stage one fieldwork was launched prior to stage two, there were no requirements imposed on completing stage one prior to stage two (upon launching stage two fieldwork). The initial qualitative participants were eligible to take part in one or both stages of quantitative research.

### Eligibility

People were eligible to take part if they had received treatment for CLL, were a citizen or permanent resident of Australia, were aged 18 years or over, and were not an employee of a pharmaceutical or medical device company.

### Quantitative online survey completion

Participants were asked to complete two online surveys on separate occasions. Each survey was approximately 30 min in duration: Stage 1: Understanding the CLL Patient Experience, and Stage 2: Understanding treatment preferences and long-term treatment goals with CLL treatment. Socio-demographic questions were presented in each survey, as were questions about disease history, treatments and care received. Stage 1 was completed between 15 July to 21 November 2022 and incorporated a patient experience BWS task and additional survey questions. Stage 2 fieldwork commenced approximately two months after the launch of Stage 1 fieldwork. Stage 2 fieldwork was completed between 13 August 2022 to 1 February 2023 and featured a DCE to develop a model that was used to explore CLL treatment preferences. The Stage 2 survey also incorporated a separate BWS task to investigate treatment goals, and additional background questions covering diagnosis, genetic markers, treatment side effects, tablet frequency preference and concomitant proton pump inhibitor (PPI) medication.

### Stage 1: Understanding the CLL patient experience

#### Best-worst scaling task: patient experience

**Fig. 1:**
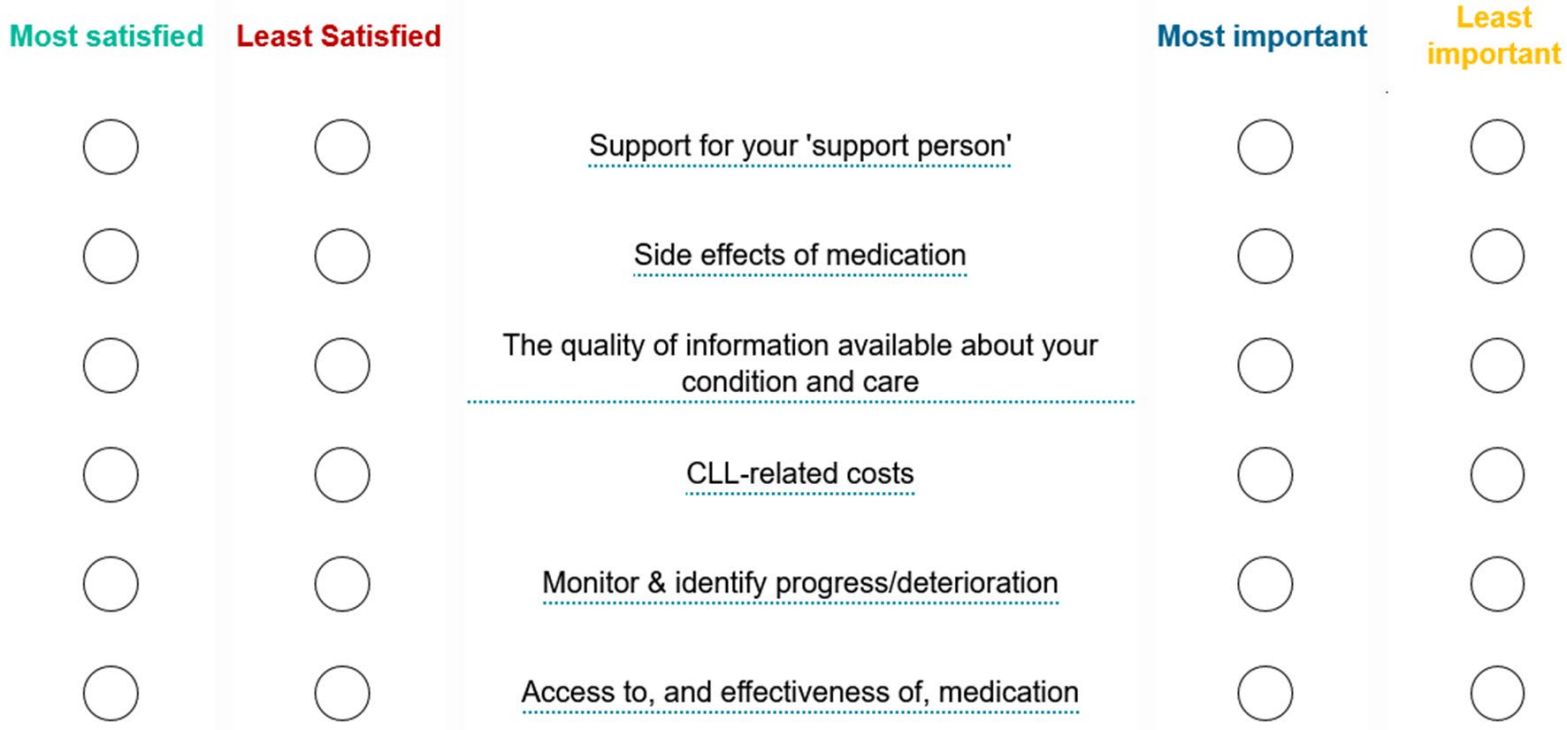
Example of patient experience BWS task scenario

This BWS exercise was designed to measure the importance and satisfaction with different aspects of the healthcare pathway. BWS is a survey method to identify priorities of items based on extremes (e.g., best / worst or most / least from a defined set of items). Participants were shown scenarios that included a subset of six items drawn from the master list of 11 Moments that Matter (MTM) statements as per Table 1 (full descriptions available in the Supplementary Information). Participants were asked to select the best and worst MTM in terms of satisfaction and importance from each of 11 scenarios. For the top four aspects of the healthcare pathway that participants had selected as the most important, however for which they had the lowest level of satisfaction, participants were asked to provide reasons for dissatisfaction and suggestions for improvements in open text entry fields. An example scenario is shown in Fig. 1.

**Table 1:** Moments that Matter statements for stage 1 understanding the CLL patient experience

1	Time to diagnosis
2	The quality of information available about your condition and care
3	Your involvement in decision making
4	The quality of your healthcare team - access to your key healthcare professional(s), consistency of care, and their communication with you and between each other
5	Treatment logistics
6	Access to and effectiveness of medication
7	Side effects of medication
8	Monitoring and identifying progress/deterioration
9	Access to other treatments/services (including a care coordinator), to support physical health, mental health, overall wellbeing (holistic approach)
10	Support for your ‘support person’
11	CLL-related costs

The BWS task was used to measure the importance and satisfaction of the different aspects of the healthcare pathway among CLL patients. This resulted in the PEI which is a combined score of the 11 BWS MTM, accounting for both satisfaction and importance (ranging from from 0 (not satisfied) to 100 (completely satisfied)) with higher scores indicating greater satisfaction. The PEI score can be used to understand the overall patient experience and identify areas of the healthcare system to target to make a positive change. Future research could also use the PEI to assess shifts in satisfaction and importance ratings and judge the success of programs, interventions and communications that are implemented to address current unmet needs.

### Stage 2: Understanding treatment preferences and long-term goals with CLL treatment

#### Discrete choice experiment (DCE) task

Participants in Stage 2 completed a DCE to evaluate treatment attributes that drive treatment preferences. DCEs are trade off experiments that involve participants comparing hypothetical treatment alternatives in a scenario and asking them to select their most preferred option. To make their choice, participants are encouraged to trade off the differing attributes of each treatment alternative according to their own unique value framework. Analyzing the observed choices using statistical models allows us to quantify the overall value of such treatments and the relative importance of each of its defining attributes. The DCE used a generic design (unlabeled) which compared two hypothetical alternatives labeled as ‘Treatment A’ and ‘Treatment B’ and an opt-out option, ‘Neither of these’ (denoting remain on current treatment). Treatments A and B were framed as alternative treatment options provided by their doctor for management of CLL. Hypothetical treatments varied in terms of seven attributes and participants were provided with detailed descriptions of the attributes and levels. The treatment attributes are similar but not identical to existing real-life treatments. Daily tablet numbers were not specified. A summary is provided in Table 2.

**Table 2:** Treatment preferences DCE: attributes, descriptions and levels

Attribute	Description	Levels	
Type and Duration of treatment	**1) Type of treatment: This can be a needle into the vein [intravenous (IV) infusion], tablets or a combination of needle (IV) and tablets.** • Treatment involving a needle into the vein (IV) is received in hospital usually every month for 6 months. • Tablets are usually taken daily at home for a fixed time or ongoing. Combination treatment involves a needle into the vein (i.e., IV in hospital every month for 6 months) plus daily tablets (for a fixed time or ongoing). **2) Duration of treatment: This can be for a fixed time or ongoing.** • Fixed time periods can include: 6 months (i.e., treatment with needle in the vein every month), daily tablets for 15 months or daily tablets for 24 months. • Ongoing treatment: daily tablets [with or without treatment with needle in the vein (IV every month for 6 months)] until CLL advances, or unacceptable side effects are experienced.	**Type of therapy**	**Total Duration of therapy**
		IV alone	6 months (fixed)
		Daily oral plus IV	15 months (fixed)
		Daily oral plus IV	24 months (fixed)
		Daily oral plus IV	Continuous (ongoing)
		Daily oral alone	6 months (fixed)
		Daily oral alone	15 months (fixed)
		Daily oral alone	24 months (fixed)
		Oral alone	Continuous (ongoing)
Average time to disease progression	**3) Average time until your CLL progresses**: This refers to the length of time during and after a CLL treatment that you live with CLL but the condition is stable/not getting worse.	**Average time until progression**	
		2 years	
		4 years	
		6 years	
		8 years	
		10 years	
Likelihood of side effects	**4) Mild to moderate side effects**: The chance of experiencing mild to moderate side effects with your CLL treatment. As a reference point, please consider the chance of you experiencing (most unacceptable side effect piped from previous question) as a mild to moderate side effect. **5) Severe side effects**: The chance of experiencing severe side effects with your CLL medication (i.e. side effects that are unacceptable and that significantly interfere with daily life. These side effects are likely to require additional treatment and/or hospitalization or may lead to having to stop that medication). Examples of severe side effects could include severe infection [e.g. pneumonia], hemorrhage (i.e. bleeding), heart issues (e.g. atrial fibrillation or heart failure)	**Mild to moderate**	
		0%	
		15%	
		30%	
		45%	
		60%	
		**Severe**	
		0%	
		10%	
		20%	
		30%	
Long-term complication risk	**6. Long-term risk**: The chance of experiencing a long-term complication with your CLL treatment. Examples of long-term complication risks could include organ damage (e.g., kidneys, liver, heart, bone marrow), secondary cancers (e.g., skin cancer), increased blood pressure.	**Long-term risks**	
		0%	
		1%	
		5%	
		8%	
Annual out-of-pocket costs	**7. Out of pocket costs**: This refers to average costs over one year that you spend due to CLL (e.g., travel, doctor costs, hospital costs). This does not include treatment costs as these are covered by the government (Medicare).	**Average out-of-pocket costs**	
		$0	
		$1000	
		$2000	
		$3000	
		$4000	

*Note* The type and duration of treatment were shown to participants in the experiment as a single attribute (of which there were 8 possible levels)

**Fig. 2:**
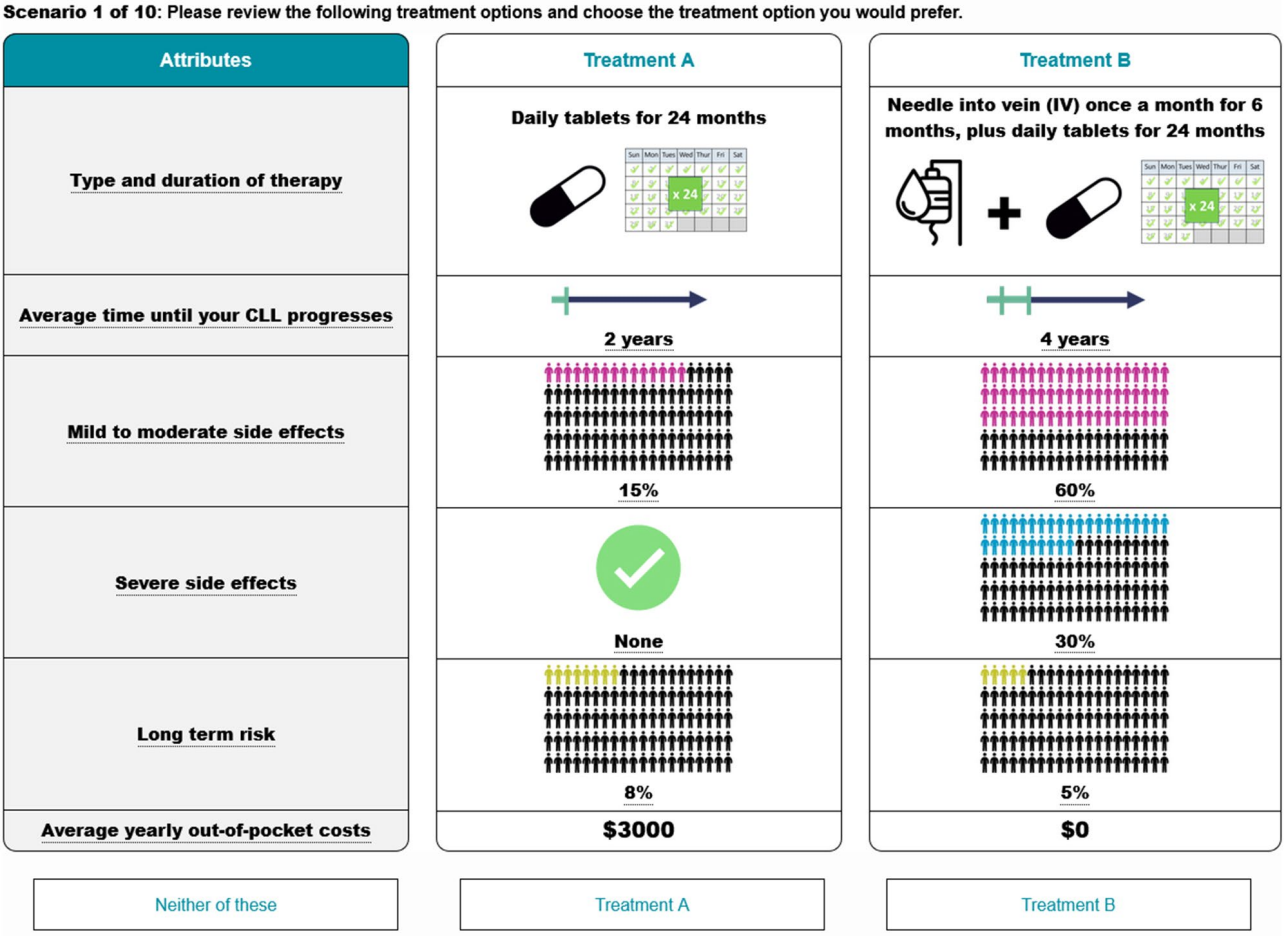
Example of treatment preferences DCE scenario

Attribute levels were organized into 80 distinct scenarios that were split into eight blocks, so that each participant was presented with 10 different choice scenarios. The combination of levels presented in the tasks were designed using a D-efficient design structure in NGene [18]. An example DCE scenario is shown in Fig. 2.

### Best-worst scaling task: long-term treatment goals

The BWS exercise was designed to understand which long-term treatment goals patients with CLL most valued. Participants were asked to consider the next 2 to 3 years and select their most important and least important treatment goals. Participants were shown 11 scenarios that included a subset of six items drawn from a master list of 11 statements as per Table 3 (detailed descriptions are in the Supplementary Information).

**Table 3:** Long-term treatment goals statements for stage 2 understanding CLL treatment goals

1	Having an income and/or work
2	Being physically healthy
3	Living a long life
4	Spending time with my family/friends
5	Pursuing my interests in life
6	Being independent
7	Feeling well emotionally
8	Avoiding hospitalization
9	Having financial wellbeing
10	Reducing my hospital/clinic visits
11	Being able to stop ongoing treatment

### Data analysis and reporting

Data quality control measures were implemented for quantitative data collection to identify false or fraudulent participants who completed the study for their own gain (e.g., to obtain a monetary benefit). Participants who finished the survey in less than 7 min (based on CaPPRe internal testing which validated that it would not be possible to complete the survey properly in under 7 min), failed the attention test, failed the DCE understanding test, were suspected duplicates or provided suspicious or nonsensical responses to open ended questions (i.e. illogical, meaningless, fake responses or directly copied from the internet) were removed during the cleaning process.

Participant demographics and disease characteristics were summarized using descriptive statistics.

#### Long-term treatment goals calculation

Data from the treatment goals BWS exercise were computed into BWS scores, resulting in a number between 1 and -1 which represent a relative ranking of the 11 items in terms of their importance. The scores for each item were calculated by finding the difference between the number of times a goal statement was chosen as most important and least important, and then dividing it by the total number of times it appeared throughout the exercise.

#### Patient experience index calculation

Data from the MTM BWS exercise were computed into BWS scores (in the same manner as described for the long-term treatment goals calculation). A novel anchoring process [15] was used to rescale best-worst scores for importance and satisfaction which were combined to form a CLL Patient Experience Index (PEI). BWS scores were calculated for each MTM by subtracting the number of times it was chosen as worst (least satisfied/least important) from the number of times it was chosen as best (most satisfied/most important), divided by the number of times it was shown throughout the task. Then the BWS scores were mapped onto a scale ranging from 0 (‘Not satisfied at all’/‘Not important at all’) to 10 (‘Completely satisfied’/’Extremely important’) describing the level of satisfaction and importance. These rescaled scores allowed direct inference of how satisfied/important each individual MTM is, rather than just their relative ranking. Rescaled scores ranged from 0 to 10 and represented the individual level of satisfaction and importance experienced. Importance BWS scores were exponentiated to create importance weights. Rescaled satisfaction scores were then weighted by importance weights, resulting in an index score ranging from 0 to 100.

#### Treatment preferences DCE task analysis

The choices observed across all participants and scenarios were used to model treatment preferences. In order to produce statistically reliable parameter estimates for small sample sizes, as with this research (*N* = 30), it is necessary to pool the responses obtained from multiple participants. As such, DCEs consist of numerous participants being asked to complete a number of choice scenarios in which they are asked to select one or more alternatives from among a finite set of alternatives. The total number of scenarios answered provide the number of observations for modeling purposes.

For the DCE analysis, attributes were re-coded for analysis. All attributes were continuously coded with the exception for duration/type of treatment which was simple effects coded (categorical), using one of the levels as a reference category (for an attribute with $l$ levels, $l-1$ new variables were created). Additionally, various attribute levels are preferred over others, impacting predicted preference share. Econometric software, Nlogit version 6, was used to model the DCE data. The model structure was consistent with the Random Utility Theory which states that decision makers compare alternative goods and services within a market and select the bundle of attributes or goods that yield the maximum utility. A mixed multinomial logit model (MMNL) was used to model the DCE data. Details of the MMNL utility function and construction of the model are available in the Supplementary Information, including a justification on why the MMNL was used in preference over the simpler, Multinomial logit model (MNL). Prior publications describe DCE analysis methods in detail [19, 20], as well as calculation of attribute importance [21–24]. Relative attribute importance (RAI) is directly associated with predicted preference share where the greater the importance weight, the more that attribute will influence predicted preference share. Additionally, various attribute levels are preferred over others, impacting predicted preference share. The RAI was calculated by finding the maximum difference in utility between the attribute levels and expressing this difference as a percentage of the sum of all maximum differences. To operationalize the DCE model and enable visualization of the results in a meaningful manner, Decision Support Systems (DSS) or ‘dashboards’ were constructed using the Shiny package in R (an open-source R package that provides a web framework for building web applications using R; RStudio, Boston MA, USA). Dashboard simulations were run to illustrate how treatment experienced patients with CLL trade off risks and benefits in making treatment choices.

## Results

Twenty-five Australian treatment experienced CLL patients completed the Stage 1 survey, and thirty patients completed the Stage 2 survey. Some participants completed both stages of research.

### Stage 1: Understanding the CLL patient experience

#### Patient characteristics

Participant demographic and treatment characteristics are shown in Table 4. Nearly two-thirds of treatment experienced patients with CLL identified as male (64%). The majority were aged 41 years or older (88%) and over half were retired (52%). Most participants lived in metropolitan areas and nearly a third were receiving second-line treatment (32%).

**Table 4:** Demographic and CLL treatment experience of patients with CLL participating in Stage 1: understanding the CLL patient experience

	Patients (*n* = 25) *n* (%)
**Sex**	
Male	16 (64)
**Age**	
31–40 years	3 (12)
41–50 years	5 (20)
51–60 years	1 (4)
61–70 years	10 (40)
71–80 years	5 (20)
81 years or older	1 (4)
**Working status**	
Working full time	6 (24)
Working part time or casual	3 (12)
Retired	13 (52)
Not working or other	3 (12)
**Location**	
Location metro/city	16 (64)
Location regional	8 (32)
Location rural	1 (4)
**Current treatment line**	
First-line treatment	7 (28)
Not currently on treatment due to achieving remission /minimal disease activity from first-line treatment	4 (16)
Second-line treatment	8 (32)
Not currently on treatment due to achieving remission /minimal disease activity from second-line treatment	2 (8)
Third or fourth-line treatment	0
Not currently on treatment due to achieving remission /minimal disease activity from third or fourth-line treatment	1 (4)
Other (e.g. being on maintenance therapy; not being on treatment due to lack of tolerability)	3 (12)
**Treatment regime**	
Targeted therapy	20 (80)
Chemoimmunotherapy	17 (68)
**Treatment experience**	
Oral medication	20 (80)
Intravenous infusion	19 (76)
Subcutaneous injection	5 (20)
Other	1 (4)
**Treatment setting**	
Public	9 (36)
Private	9 (36)
Combination of public and private	7 (28)

*Note*^**a**^ there were no specific CLL treatments available in Australia at the time of the study that are administered by SC infusion

More participants reported having received targeted therapy (80%) than chemoimmunotherapy (68%). Treatment experience with oral medication or intravenous (IV) therapy was equally common. Subcutaneous (SC) injection responses reflect patients indicating that a CLL treatment was injected under the skin. There were no specific CLL treatments in Australia that are administered by SC infusion at the time of the study, and so this response rate may reflect a patient misunderstanding between SC and IV administration. In addition, concurrent treatments such as growth factor or anti-emetics could be represented. The number of participants treated in exclusively public or private settings was evenly distributed with 28% having been treated in a combination of public/private settings. The greatest out-of-pocket cost to patients with CLL was private healthcare insurance cover, with a mean cost of $3,147 annually, followed by visits to healthcare professionals and medication costs. Nearly two-thirds of treatment experienced patients were supported by patient support organizations (64%) and just over half of the participants have made use of reminder services for their treatment, prescriptions, and appointments (52%). Eight percent had a care coordinator.

### Best-worst scaling: patient experience

**Fig. 3:**
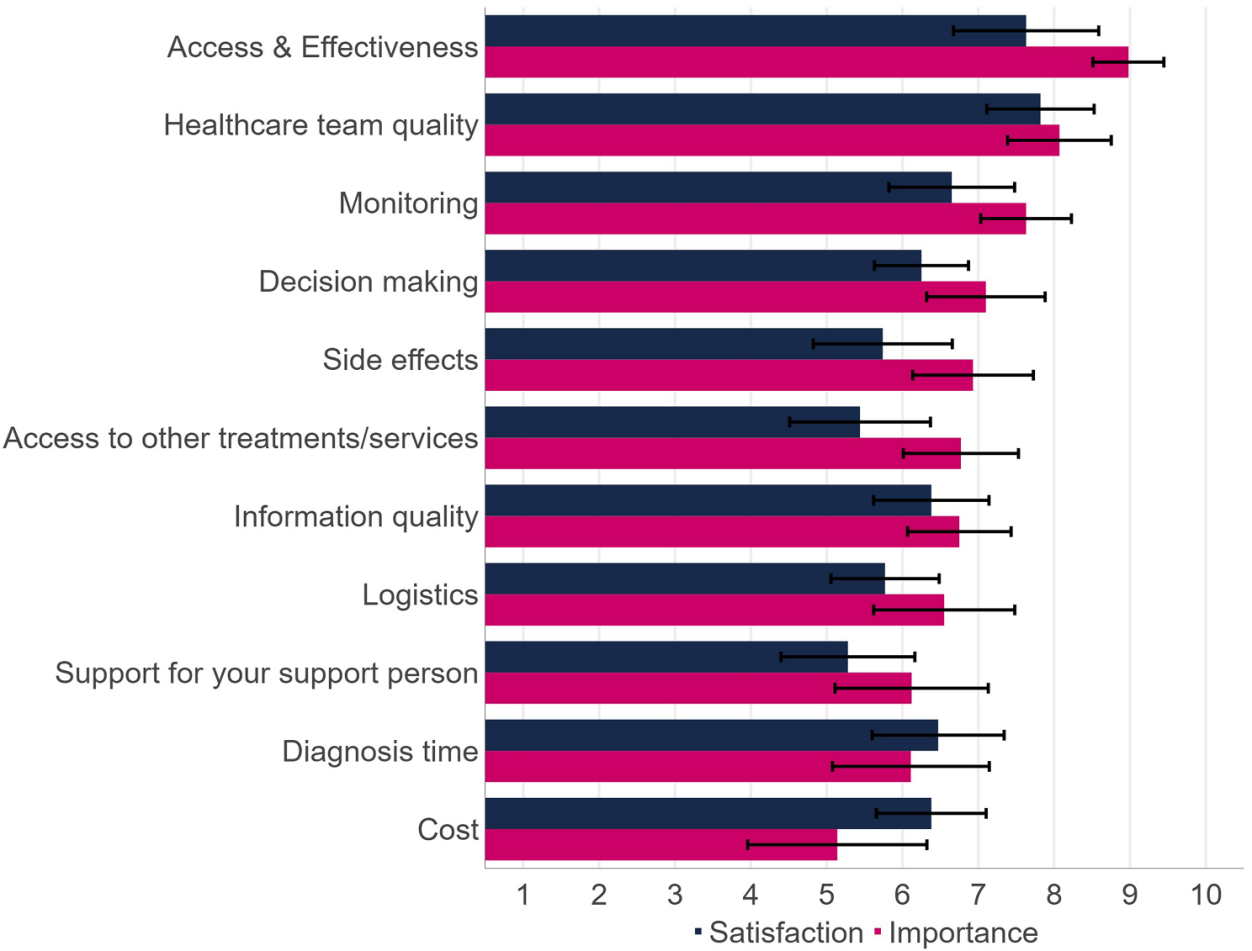
Importance and satisfaction scores (rescaled to score out of 10) with 95% confidence intervals

All MTM were found to be at least somewhat important, with none scoring below 5 out of 10. The top three MTMs in terms of importance to patients were ‘Access to and effectiveness of medication’ (8.98), ‘Healthcare team quality’ (8.07), and ‘Monitoring and identifying progress or deterioration’ (7.63) (Fig. 3).

Participants noted dissatisfaction with access to medication, particularly restrictions around lines of therapy, and access to clinical trials for those outside metropolitan areas. Participants were least satisfied with ‘Support for your support person’ (5.28), noting that their carer received limited emotional support, especially during the COVID-19 pandemic. ‘Access to other treatments/services’ (5.44), ‘Side effects of medication’ (5.74) and ‘Treatment logistics’ (5.77) also had lower levels of satisfaction. Examples included a lack of services to help with lifestyle changes or provision of complementary therapies. Satisfaction levels were lower than importance levels on all MTM except ‘Diagnosis time’ and ‘CLL-related costs’.

**Fig. 4:**
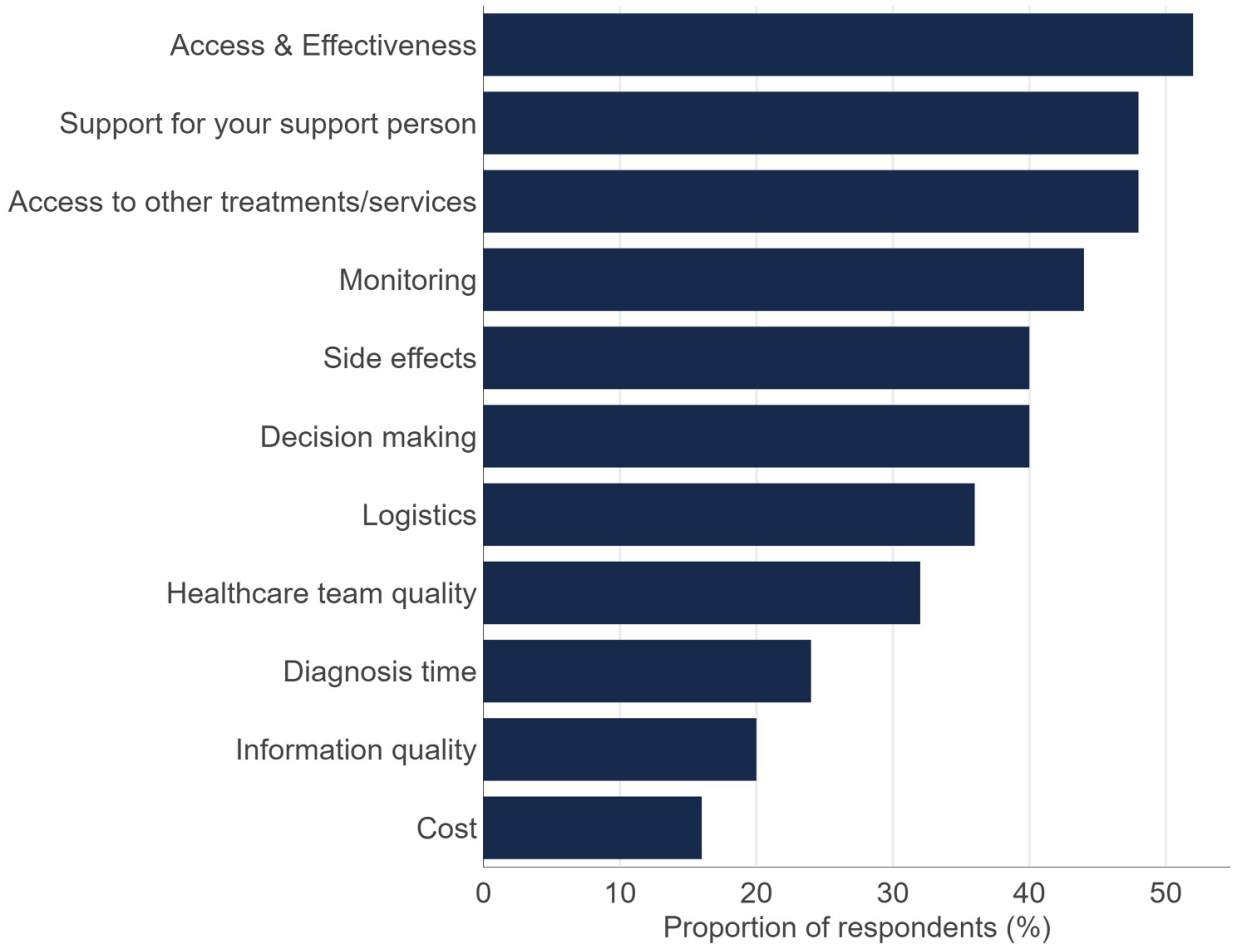
Top 4 Most important and least satisfied moments that matter (MTM)

Combining the most important and least satisfied domain for each patient resulted in the top 4 most important and least satisfied MTM shown in Fig. 4: ‘Access to and effectiveness of medication’ (52% of patients reported this as one of their top 4 ‘least satisfied but most important’), ‘Support for your support person’ (48%), ‘Access to other treatment/support services’ (48%) and ‘Monitoring and identifying progress or deterioration’ (44%). Other treatments or services that were particularly important to participants included provisions to support lifestyle changes (such as diet, exercise and sleep) and access to advocacy groups that can provide holistic support for patients with CLL. Participants who had the monitoring MTM in their top four for high importance/low satisfaction were mostly dissatisfied with the availability of tools to help them track physical or mental health changes; they would like to be able to easily view details of their progress such as changes to blood test results over time.

#### The patient experience index

The median PEI score (i.e., overall satisfaction score) for treatment experienced patients with CLL was 66.2 out of a possible 100 (standard deviation 14.02). To review the PEI score drivers, readers can access the online dashboard at https://cappre.shinyapps.io/PEI_Dashboard_CLL_Tx_AUS/.

### Stage 2: Understanding treatment preferences and long-term goals of CLL treatment

#### Patient characteristics

The demographic characteristics of treatment experienced patients who completed the Stage 2 survey are shown in Table 5. The demographics were similar to the patients in Stage 1, although more Stage 2 participants were retired. At the time of Stage 2 survey completion, treatment experienced patients were either on first (43%) or second-line (48%) treatment.

**Table 5:** Demographic characteristics of patients participating in stage 2: CLL Treatment preferences and Treatment goals

	Patients (*n* = 30) *n* (%)
**Sex**	
Male	18 (60)
**Age**	
31–40 years	2 (6.67)
41–50 years	2 (6.67)
51–60 years	2 (6.67)
61–70 years	10 (33.33)
71–80 years	12 (40)
81 years or older	2 (6.67)
**Working status**	
Working full time	1 (3.33)
Working part time or casual	5 (16.67)
Retired	21 (70)
Other	3 (10)
**Location**	
Location metro/city	17 (56.67)
Location regional/rural	13 (43.33)

Just over 70% of participants were on monotherapy, most frequently receiving ibrutinib (46.7%), followed by acalabrutinib (26.7%). There were no participants on zanubrutinib. Among the 30% of participants who were on combination therapy, venetoclax plus obinutuzumab (66.67%) was the most common combination. Nearly one-quarter of the sample (23.3%) reported awareness of a genetic marker for a molecular or cytogenetic abnormality associated with their CLL. The remainder of the participants did not know if they had a genetic marker (26.7%) or were unsure (50.0%). Specific markers stated by participants included 13q deletion, *TP53* mutation, unmutated immunoglobulin heavy chain (*IGHV*), mutated *IGHV* and 6q deletion.

When asked about frequency of oral medications, there was a strong preference among participants for taking three tablets once a day (63.33%) versus one tablet twice a day (30%). Only three patients provided feedback on comfort levels in changing their PPI due to CLL treatment requirements with a mean score of 5.33 on a scale of 1 (not at all comfortable) to 10 (completely comfortable).

#### Discrete choice experiment

The results from the best fitting MMNL model based on the 30 treatment experienced patients are shown in Table 6. The table exhibits the parameter coefficients, standard errors (SE), T-ratio, and associated p-value. For the random parameter coefficients, the estimated mean value is reported. The distribution from which the IV and FIXED parameters were drawn was the constrained normal distribution, while all other random parameters were drawn from the triangular distribution with the variance parameter constrained to 1.5 times the mean.

**Fig. 5:**
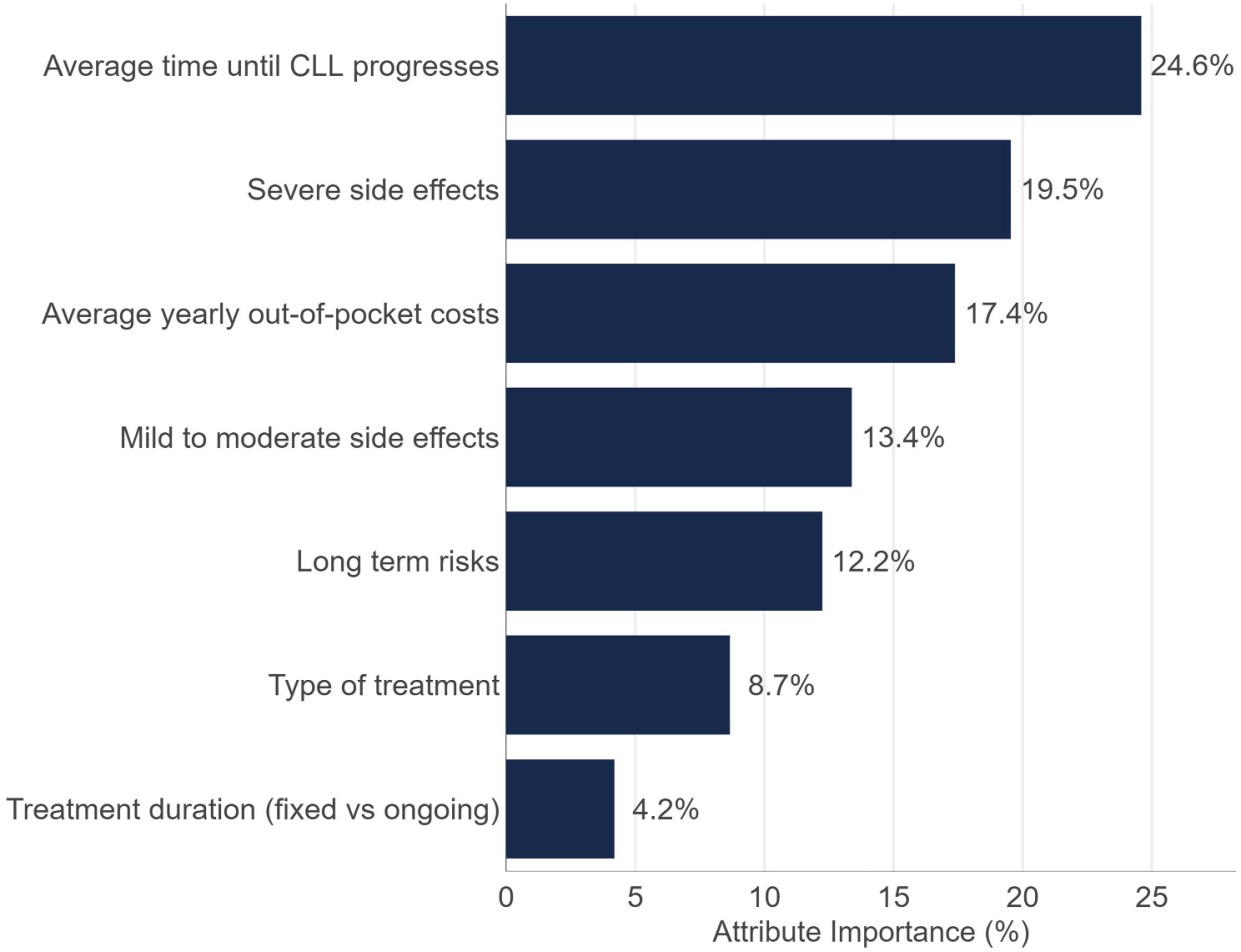
CLL relative attribute importance

The relative attribute importance is depicted in Fig. 5. Attribute importance is directly associated with predicted preference share where the greater the importance weight, the more that attribute will influence predicted preference share. The relative attribute importance (RAI) sums to 100%. All attributes significantly predicted choice and were important to patient decision making in the experiment.

**Table 6:** DCE Model results

Random parameters	Parameter symbol	β Co- efficient	SE	T-ratio	*p*-value
Preference for new treatment plan	NEW	3.626	0.831	4.36	< 0.01
Type of treatment involves IV (Reference category: Treatment does not involve IV)	IV	-0.538	0.218	-2.47	0.013
Treatment duration is fixed (Reference category: Treatment duration is ongoing)	FIXED	0.261	0.154	1.69	0.091
Average time to disease progression (Range: 2 years – 10 years)	PFSC	0.382	0.087	4.40	< 0.01
Likelihood of mild to moderate side effects (Range: 0 – 60%)	MILDC	-0.028	0.008	-3.65	< 0.01
Likelihood of severe side effects (Range: 0 – 30%)	SEVC	-0.081	0.018	-4.49	< 0.01
Long-term risk of complications (Range: 0 – 8%)	LONGC	-0.190	0.051	-3.75	< 0.01
Average annual out-of-pocket costs (Range: $0 - $4000)	COSTC	-0.001	0.000	-3.79	< 0.01

*Notes* β Co-efficient and SE results rounded to three decimal places. The magnitude of the coefficient indicates the strength of the predictive value of each attribute. For the random parameter coefficients, the estimated mean value is reported

*Abbreviations* SE, standard error; DCE, discrete choice experiment

When choosing between theoretical CLL treatments in the DCE ‘Average time to disease progression’ resulted in a statistically significantly positive coefficient (β = 0.382) and this attribute had the greatest RAI of 24.6%. This indicates that as PFS increases, so do preferences for that treatment. Further to this, the DCE model results demonstrate that a new treatment (Treatment A or Treatment B) was preferred over existing treatment (the opt-out ‘neither of these treatments’). This is shown by the positive coefficient of the random parameter ‘Preference for new treatment plan’ (β = 3.626) where participants chose a new treatment (i.e. Treatment A or Treatment B) compared to the opt-out of ‘neither of these treatments’ when all other attributes were constant (Table 6).

DCE results also demonstrated a patient preference for oral administration over IV (seen by the negative and significant coefficient for ‘Type of treatment involves IV’, β=-0.538, *p* < 0.05) with RAI for ‘Type of treatment’ 8.7%. The impact of route of therapy was greater than that of treatment duration (i.e. fixed vs. continuous, RAI 4.2%). Relative importance of each attribute is shown in Fig. 5.

There was a slight but not significant preference for fixed versus continuous therapy (i.e., β = 0.261; *p* < 0.10). Patients preferred treatments that had lower levels of barriers and risks with ‘Mild to moderate side effects’ (β=-0.028), RAI 13.4%, ‘Severe side effects’ (β=-0.081), RAI 19.5% ‘long-term complication risks’ (β=-0.190), RAI 12.2% and ‘average annual out-of-pocket costs’ (β=-0.001) RAI 17.4% all yielding significantly negative coefficients. As the levels for these attributes increase (i.e. increased mild to moderate side effects, more severe side effects or higher costs), preferences for the treatment decrease.

#### Dashboards and simulations

A simulation model has been developed using the DCE treatment preferences data (https://cappre.shinyapps.io/CLL_Goals_Preferences/_w_110142e1/).

The dashboard supports the visualization of the model and enables users to simulate scenarios based on changes to the attributes of hypothetical therapies. Illustrative simulations examining the impact of adjustment to treatment attributes on patient preferences are presented in the Supplementary Information. Five simulations examined ongoing oral versus fixed duration IV treatment, treatment benefit trade off, treatment risk trade off, impact of increased out-of-pocket costs and impact of IV administration as detailed in Table 2.

### Best-worst scaling: long-term treatment goals

**Fig. 6:**
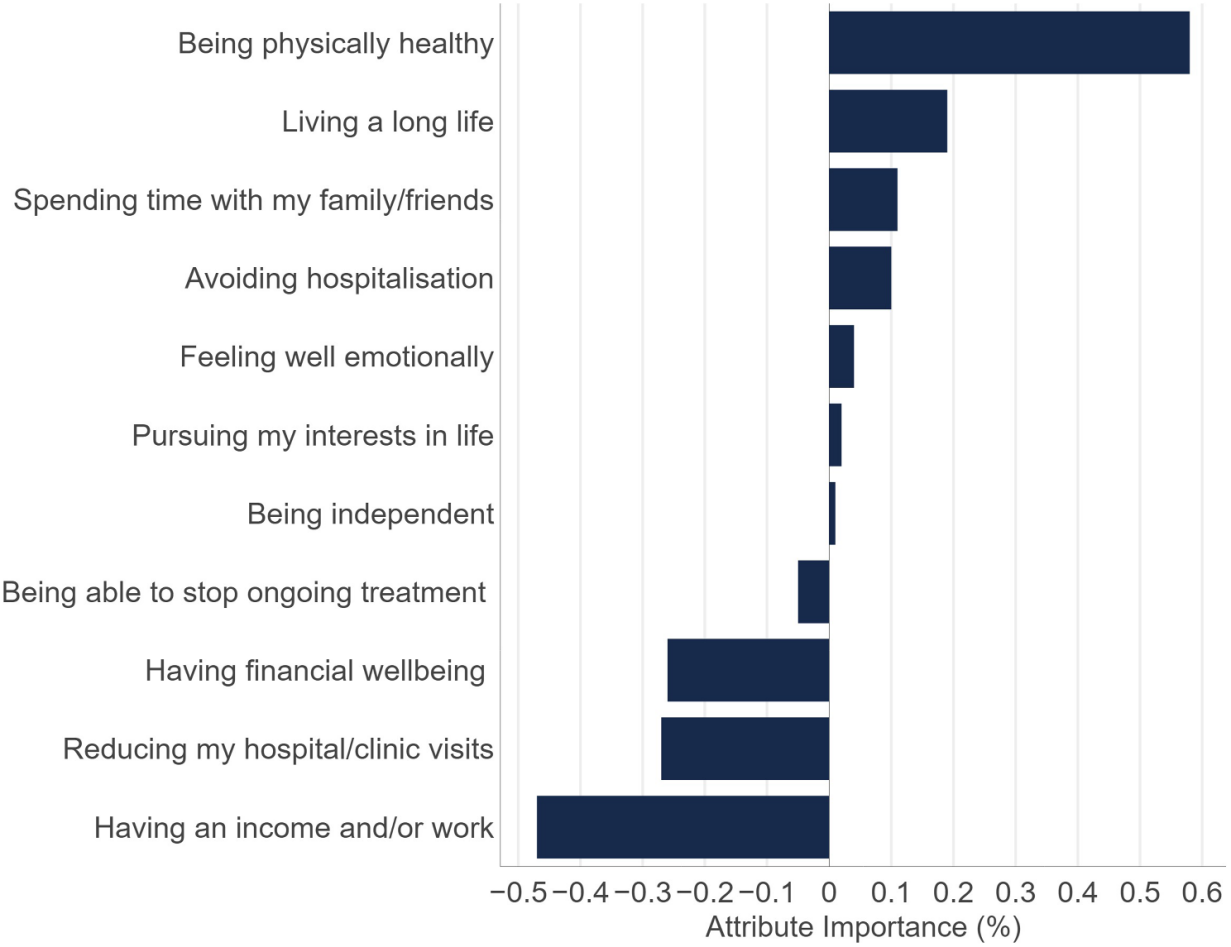
CLL BWS treatment goals

Results from the BWS task found that the most valued long-term treatment outcome for treatment experienced patients living with CLL was the ability to be physically healthy (Fig. 6). This was followed by the prospect of living a long life, spending time with family and friends, and avoiding hospitalization. The least important outcomes included having an income and/or work, reducing hospital and clinic visits and financial wellbeing.

## Discussion

Findings from this research increase our understanding of the healthcare goals and treatment experiences of Australians living with CLL.

### Rationale for selection of experiments

Incorporating consumer values into health outcomes is becoming more central to healthcare design [11] and is known as patient value mapping [21, 22, 25]. The patient value framework is established using techniques such as DCEs, which directly measure how patients make decisions by deriving relative importance of specific components of a treatment using an experiment. This is achieved by showing patients a set of hypothetical competing treatment alternatives and asking them to select their most preferred option, according to their own unique values [12]. DCEs are used by many fields of medicine to understand and model the trade-offs and preferences revealed by the choices that people make [21–23, 26, 27]. The underlying assumption is that choices made in a DCE reflect patient treatment preferences and can indicate perceived overall value of a treatment. A recent systematic review of DCEs in cancer patients called for additional research in this area in order to create a robust evidence base [27].

### Patient experience

The results indicate that patients most value the ability to access effective medication and a high-quality healthcare team. The median PEI score was 66 out of 100, and analysis of drivers of the PEI score found that no MTM rated high on importance but low on satisfaction, which suggested that patients are largely satisfied with the aspects of treatment and care that are important to them. The top four least satisfied but most important results reflect areas in which there is scope to improve patient care and the treatment experience by implementation of programs or system changes for treatment experienced patients living with CLL. Specifically, this includes access to, and effectiveness of medication, followed by support for the carer and access to other treatments and services.

### Access to effective treatment

Low satisfaction with access to effective treatment may be driven by frustration that subsidized access to novel medication is restricted, even if their healthcare professional (HCP) considers it the best fit. For example, until recently the BTKi were registered but not subsidized for first-line therapy in Australia, despite the fact that those treatments have been available in this line of therapy for several years in different geographies. Further, patients were disappointed that having comorbidities or living in rural locations could exclude them from clinical trials. Some patients also perceived a gap in their ability to access other treatments and services such as complementary therapies or support groups for holistic care.

### Support and monitoring

Exploring differences in the magnitude of satisfaction and importance (i.e., key gaps), revealed that there may be opportunities for improvement of communication regarding monitoring of patients with CLL. Patient scores for monitoring of disease reflect a desire to be able to track their own physical or mental health measurements over time, with some patients mentioning that ‘tracking apps’ would assist with this. However, CLL is an indolent malignancy where rapid progression is uncommon, therefore very frequent monitoring is not required. The high importance but low satisfaction with ‘Support for my support person’ indicates that there is opportunity to improve the level of inclusion and emotional support for carers. This could take the form of counseling and peer support services, and educational material that is specifically designed for carers. The COVID-19 pandemic restrictions also impacted the ability of patients to discuss results of monitoring, and for their support people to receive emotional support.

### Disease progression and side effects

Consistent with other studies [28, 29], patients in Stage 2 of this study prioritized a treatment that has the longest PFS time and lower chance of severe side effects or complications. In our DCE, average time to disease progression was the most important treatment attribute (accounting for 24.6% of decision making), followed by risk of severe side effects and high out-of-pocket costs. The importance of treatment attributes related to safe and effective therapy were unsurprising. These findings from our sample aligned with those from a US study of 220 patients with CLL and 151 clinicians demonstrating a large impact of increasing the chance of two years PFS from 75 to 90% [29]. Increasing the average time to disease progression from six years to seven years on a hypothetical treatment resulted in a greater preference share for the treatment option providing a longer PFS in the model simulation (60% versus 40%). Simulations also demonstrated that the potential for severe side effects holds weight in patients’ treatment decisions. Examples given were severe infection, hemorrhage or heart issues as outlined in Table 2. When efficacy and side effects risk was held constant between therapies, other attributes of therapy, as explained in the next section, became more important to participants.

### Method and frequency of administration

In common with the current study, a DCE carried out in Italy found that both patients and clinicians preferred orally administered therapy over intravenous therapy, however the duration of therapy was less relevant [28]. Patients in the Italian study scored the mode and timing of treatment administration more highly (RI 20%) than clinicians (RI 12%) [28]. Further, patients in our study preferred once daily oral dosing over a twice daily option. In addition, a global survey of perspectives of real-world patients with CLL found that oral medication is preferred to IV treatment for high risk disease [30]. Taken together, this indicates that a targeted treatment approach that can be prescribed as a ‘once daily oral alone’ will hold advantages compared to treatments with an intravenous component, all other factors being equal. It has been previously reported that some patients with CLL prefer time-limited therapy compared to oral continuous therapy [5, 30], however in our model, treatment type (for example ongoing oral treatment without an intravenous component) held more relative importance than treatment duration (fixed or ongoing).

### Out-of-pocket costs

Out-of-pocket costs had a high relative attribute importance, ranking after disease progression and severe side effects. These were viewed in the context of payments specifically associated with CLL management including travel, doctor, hospital and treatment costs.

### Long-term treatment goals

Our sample of treatment experienced CLL patients most valued a treatment that would enable them to achieve their goals of being physically healthy, living a long life, spending time with family and friends, and avoiding hospitalization. The treatment goals stated as less important included having an income and/or work, reducing hospital and clinic visits, and having financial wellbeing. Within the BWS task, patients viewed treatment goals in a broad context (e.g., considering all aspects that may be related to a specific goal). Treatment goals relating to financial wellbeing and having work were relatively less important when considering treatment outcomes, possibly due to the older age of participants included, and that many were retired. It is also possible that this result was due to a skew in our sample toward a population with higher means and education able to respond to an online survey. The DCE results showed that when looking at treatment attributes at a more granular level, patients will choose a treatment that will give them longer and better quality of life, consistent with the BWS findings.

### Patient-centric care

The impact of concomitant treatment should not be underestimated when discussing preferences with patients, as it has the potential to affect the quality of life. An example of this is a large proportion of CLL patients who are concurrently taking PPIs; up to 60% of the entire CLL patient population in Australia [31]. Our study indicates that patients are not comfortable with altering concomitant PPI treatment, however this score should be interpreted with caution due to the small number of responses.

Some of the newer therapies which are now available in Australia for patients with relapsed or refractory CLL have relatively comparable efficacy and safety profiles but differ in other attributes. The focus of our study aligns with recent trends for hematologists to have ongoing conversations with patients to explore treatment goals and treatment preferences [32, 33]. A better understanding of patient preferences across treatment attributes might have a beneficial impact on adherence to therapy and patient satisfaction. It is important to provide patients with opportunities to be involved in shared decision making which has potential to improve outcomes in CLL [33, 34].

### Strengths and limitations

The current study is limited in its representativeness due to the nature of the study design and small sample size. Participants needed to be willing and able to complete the reasonably demanding online experiment which required digital literacy and educational or social advantages that do not reflect the diversity of all people living with CLL. Another potential limitation is that the patients who were involved in this research may overall be more engaged and thoughtful concerning their CLL management compared to the wider CLL community due to their apparent commitment in engaging and completing research surveys. Further, the survey instruments were only available in English. The study was limited to participants in Australia, so the findings may not be generalizable to other countries. Although the treatment attributes evaluated in the BWS exercises and DCE were derived from initial qualitative interviews, literature, and expert opinion, it is possible that the study did not include some attributes that participants considered important. Finally, the nature of the study did not allow for any evolution in patient preferences over time (such as before and after CLL treatment).

## Conclusions

The patient voice within CLL is critical to informing patient centered care, both in terms of their experience with the disease and their choice of therapies. Insights from this research can help support decisions that will enhance the treatment experience of patients with CLL. An important focus is receiving effective safe therapies that will provide them with longer, healthier lives. In an era of novel, targeted therapies, consideration of other attributes, such as once daily dosing, oral medication, toxicity and quality of life, duration of therapy, and access to support services become more prominent and may affect patient treatment choices. Discussion about patient preferences and small adjustments within the treatment pathway have the potential to improve the patients’ healthcare experience and ultimately enhance their treatment outcomes.

### Electronic supplementary material

Below is the link to the electronic supplementary material.

Supplementary Material 1

Supplementary Material 2

Supplementary Material 3

## Data Availability

The datasets used and/or analyzed during the current study are available from the corresponding author on reasonable request.
