# Supplementary material for: Understanding the experience, treatment preferences and goals of people living with chronic lymphocytic leukemia (CLL) in Australia
Source: BMC Cancer. 2024 Jul 11;24:831. doi: 10.1186/s12885-024-12589-9 (PMC11241996; doi:10.1186/s12885-024-12589-9)
Supplement: Supplementary file 1 — Supplementary Material 1 [file 12885_2024_12589_MOESM1_ESM.docx]

# Understanding the experience, treatment preferences and goals of people living with chronic lymphocytic leukemia (CLL) in Australia

# Supplementary Information

## Best-worst scaling task statements and descriptions

**Table S1 Moments that Matter (MTM) descriptions for Stage 1 Understanding the CLL patient experience**

|  | **MTM** | **Description** |
| --- | --- | --- |
| 1 | Time to diagnosis | - The length of time from developing symptoms through to being diagnosed - whatever this looked like for you. |
| 2 | The quality of information available about your condition and care | - Having clear, concise, relevant information in a format that works for you (e.g., provided to you by your healthcare team/online/Apps/podcasts). |
| 3 | Your involvement in decision making | - How involved you are in decisions about your treatment and care, e.g., when selecting specific medication and/or when developing a treatment plan |
| 4 | The quality of your healthcare team - access to your key healthcare professional/s, consistency of care, and their communication with you and between each other | - Suitable access to your key healthcare professional (e.g., haematologist), at regular intervals that you feel are most beneficial to you or in acute situations where urgent access is required. - Being able to see the same trusted healthcare professional/s on-going for your treatment and care. - How well your needs are met in any interactions with your healthcare team (including doctors, nurses, care coordinators). - The extent to which different members of your healthcare team (e.g., haematologist/GP/clinical nurse specialist) communicate with each other about your condition and care. They may be healthcare professionals within the same service or in different services. |
| 5 | Treatment logistics | - The broad impact that following a treatment and care plan has on you., i.e., day-to-day difficulties of arranging and attending treatment sessions. |
| 6 | Access to and effectiveness of medication | - Your access to medication for your condition. - How effective the medication prescribed by your healthcare professional/s is in treating your condition. |
| 7 | Side effects of medication | - Side-effects you may experience from medication prescribed by your healthcare professional/s. |
| 8 | Monitoring & identifying progress / deterioration | - The ability to monitor day-to-day and long-term changes in your physical and overall wellbeing, for yourself, and by your healthcare professionals (e.g., pain, fatigue) and adjustments to treatment and care based on this. |
| 9 | Access to other treatments/services (including a care coordinator), to support physical health, mental health, overall wellbeing (holistic approach) | - Other services could include seeing a psychologist or exercise physiologist. Complementary treatments could include acupuncture, massage, mind-body techniques, and management strategies for increased wellness (e.g., access to dietitians/physiotherapists/occupational therapists/psychologists). - Having someone who is assigned to you (e.g., social worker or peer support worker) to help you navigate the healthcare system and offer emotional support and guidance. |
| 10 | Support for your ‘support person’ | - Information/websites specifically for significant others (e.g., spouse, partner, friend etc) and support groups where family members/friends can talk with others in similar situations. |
| 11 | CLL-related costs | - The overall impact that having CLL has on your financial wellbeing, e.g., how much you are out-of-pocket, and the impact of loss of income. |

**Table S2 Treatment goals descriptions for Stage 2 Understanding the value of long-term goals with CLL treatment**

|  | **Goal Statement** | **Description** |
| --- | --- | --- |
| 1 | Having an income and / or work | - Not being restricted in my ability to work or earn an income due to difficulties that arise from CLL |
| 2 | Being physically healthy | - Being physically fit and healthy (i.e., ability to exercise and participate in physical activities) |
| 3 | Living a long life | - Having a long life expectancy despite having CLL |
| 4 | Spending time with my family / friends | - Interacting / having personal relationships with my family and friends (e.g., socialising, doing things together) |
| 5 | Pursuing my interests in life | - Engaging in my hobbies (e.g., travelling, going out to restaurants) |
| 6 | Being independent | - Having autonomy (i.e., not having to rely on others) |
| 7 | Feeling well emotionally | - Feeling happy and positive; reduced concern / worry that arises from CLL (e.g., fear for the future) |
| 8 | Avoiding hospitalization | - Staying out of hospital [arising from CLL complications (e.g., infections)] |
| 9 | Having financial wellbeing | - Not feeling pressured financially due to personal costs associated with CLL (e.g., doctor fees, travel to and from hospital / clinic, parking at hospital / clinic) |
| 10 | Reducing my hospital / clinic visits | - Reducing the need for me to travel to the hospital / clinic (e.g., to have treatment or to see my specialist) |
| 11 | Being able to stop ongoing treatment | - Being able to stop having CLL treatment on an on-going / continuous basis |

## Discrete Choice Experiment Analysis methods

For the DCE analysis, attributes were re-coded for analysis. All attributes were continuously coded with the exception for duration/type of treatment which was simple effects coded (categorical), using one of the levels as a reference category (for an attribute with 𝑙 levels, 𝑙 − 1 new variables were created).

Econometric software, Nlogit version 6, was used to model the DCE data. The model structure was consistent with the Random Utility Theory (RUT), which states that decision makers compare alternative goods and services within a market and select the bundle of attributes or goods that yield the maximum utility (i.e., the participant is a utility maximiser). It proposes that overall utility, 𝑈𝑛𝑠𝑗, for alternative 𝑗 by participant 𝑛 in choice situation 𝑠, is expressed as the sum of the observable component, 𝑉𝑛𝑠𝑗, and a random component 𝜖𝑛𝑠𝑗, as follows: 𝑈𝑛𝑠𝑗 = 𝑉𝑛𝑠𝑗 + 𝜖𝑛𝑠𝑗

##### Explanation of multinomial logit model and mixed multinomial model

**Multinomial logit model**

The simplest discrete choice model, the multinomial logit model (MNL), is often used to estimate the 𝑉𝑛𝑠𝑗 component of the utility equation as a function of the alternative’s defining attributes. It takes the following form, where 𝛽𝑗𝑘 is the parameter coefficient for attribute 𝑘 of alternative 𝑗, and 𝑥𝑛𝑠𝑗𝑘 is the data variable indicating the level shown for attribute 𝑘 of alternative 𝑗 in choice scenario 𝑠 for participant 𝑛.

$$V_{nsj}=\sum_{k=1}^{K} \beta_{jk}x_{nsjk}$$

The MNL model structure relies on certain assumptions that impose restrictive conditions on its behavior. Firstly, it assumes that the error terms (𝜖𝑛𝑠𝑗) are independent and identically distributed (IID) of extreme value type 1 (EV1). This leads to further restrictions around the independence of observed choices and independence of irrelevant alternatives (IIA), where the relative probabilities of two alternatives being chosen is not affected by the inclusion or exclusion of other alternatives. Furthermore, the parameter coefficients (𝛽𝑗𝑘) are assumed to be invariant across the sample. This means that it limits itself to only one set of parameter coefficients (or parameter weights) to describe the trade-off behaviour of all survey participants combined (i.e., homogeneity of preferences).

**Mixed Multinomial logit model**

Since we cannot expect all patients to have similar treatment preferences, the homogeneity assumption is not appropriate. Therefore, the more advanced mixed multinomial logit model (MMNL) was used to model the DCE data. The MMNL structure allows greater flexibility in the model coefficients such that it can account for preference heterogeneity between participants and relax the restrictive assumptions around the IID error terms and independence of observed choices. It achieves this additional level of accuracy by allowing the parameter coefficients to be random variables drawn from a pre-specified distribution. This means that the set of parameter estimates may vary between each individual participant such that the model definition includes a 𝛽𝑛𝑗𝑘 which is specific to participant 𝑛.

$$V_{nsj}=\sum_{k=1}^{K} \beta_{njk}x_{nsj}$$

Point-estimates for the parameter coefficients 𝛽̂𝑗𝑘 are obtained by estimating the average value over the 𝑛- sets of participant-specific coefficients. These values are used in reporting and analysis.

Statistical significance was determined by the 𝑝 < 0.05 criteria and the adjusted McFadden Pseudo R- squared, which accounts for the number of parameters in a model, was used to assess model fit.

## Decision Support System (DSS) / DASHBOARD

Attribute importance is directly associated with predicted preference share whereby the greater the importance weight, the more that attribute will influence predicted preference share. Additionally, various attribute levels are preferred over others, impacting predicted preference share. For each treatment alternative, the predicted preference share is expressed as the chance (%) of it being chosen by the decision maker (i.e., patient).

To operationalise the DCE model in a manner that is meaningful, a Decision Support System (DSS) or ‘dashboard’ for the DCE models was built to enable visualisation of the model results. The dashboard enabled the user to perform ‘what if’ scenarios based on hypothesised changes to the therapies. Users of the DSS may manipulate the levels of the treatment attributes to view the preference share and resulting predicted market share for CLL treatment. The online dashboard was constructed using the Shiny package in R (Shiny is an open source R package that provides a web framework for building web applications using R).

## DCE Simulations

### Simulation 1: ongoing oral daily or fixed duration IV treatment

In this example levels for Treatment A were set to reflect the likely attributes of an ongoing daily oral targeted therapy: ‘*Average time to disease progression’*: 6 years, ‘*Likelihood of mild to moderate side effect*s’: 30%, ‘*Likelihood of severe side effect*s’: 10%, ‘*Long-term complication risk’*: 1% and ‘*Average yearly out of pocket costs’*: $1000.

Treatment B was set to reflect the likely attributes of an intravenous chemotherapy treatment administered every month for six months. These levels included: ‘*Average time to disease progression’*: 6 years, ‘*Likelihood of mild to moderate side effect*s’: 60%, *‘Likelihood of severe side effects’*: 30%, ‘*Long-term complication risk’*: 8% and ‘*Average yearly out of pocket costs’*: $1000. As shown in Figure S1 this scenario yielded a strong preference share for Treatment A (i.e., ‘ongoing daily oral alone’ targeted therapy; 96.6%) with only a very small preference share for Treatment B (i.e., IV, 6-month fixed therapy; 1.3%) and opt-out (stay on current treatment; 2.1%).

Figure S1 CLL Treatment preferences DCE Simulation 1


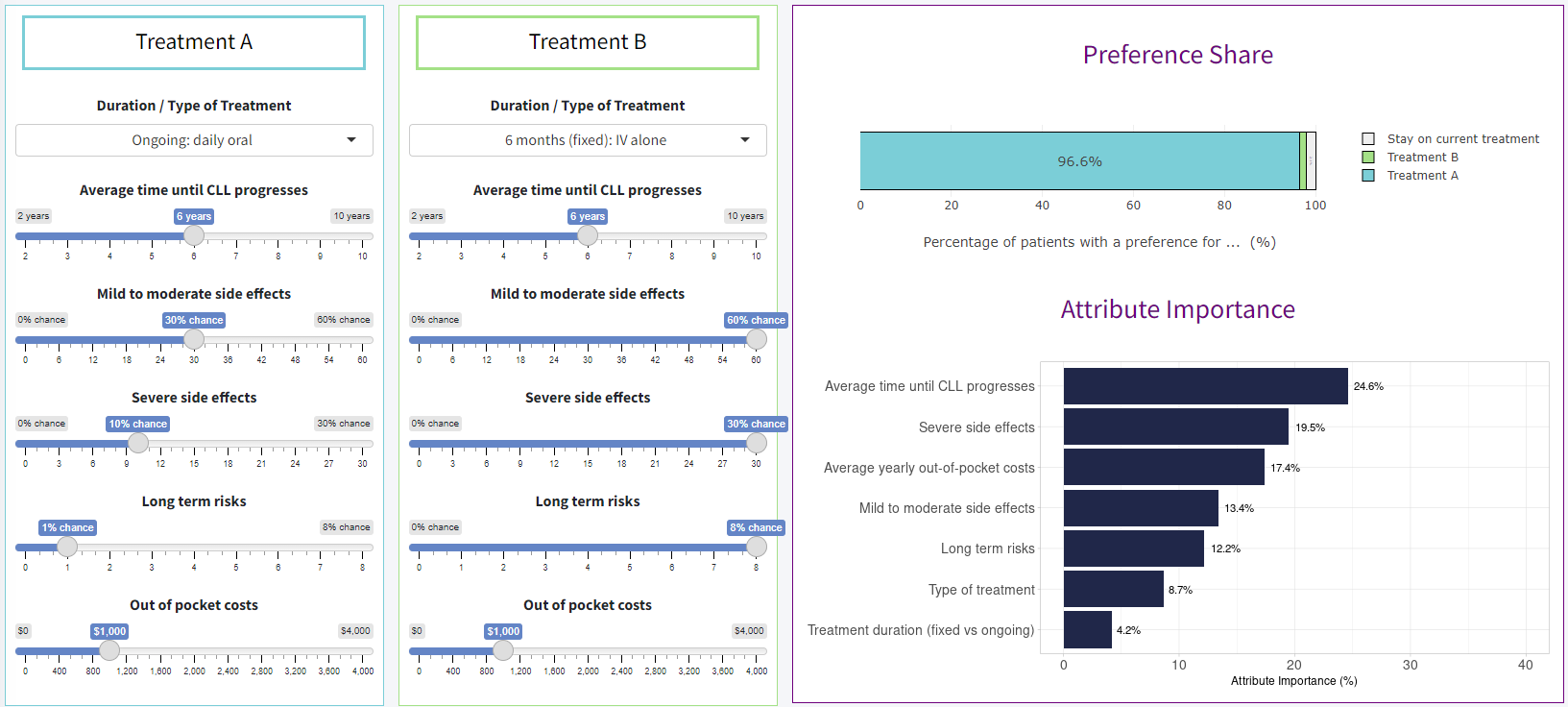


### Simulation 2: treatment ‘benefit’ trade off

In Simulation 2, we altered the most important treatment benefit attribute ‘*Average time to disease progression’*, from 6 years to 7 years for Treatment A; all other attributes for Treatment A and Treatment B were set to match the ongoing daily oral treatment from Simulation 1. This scenario demonstrated how treatment experienced CLL patients trade off the risks and benefits. As seen in Figure S2, in this case, preference share for the treatment with longer PFS was 58.9% (7 years) versus 40.2% (6 years).


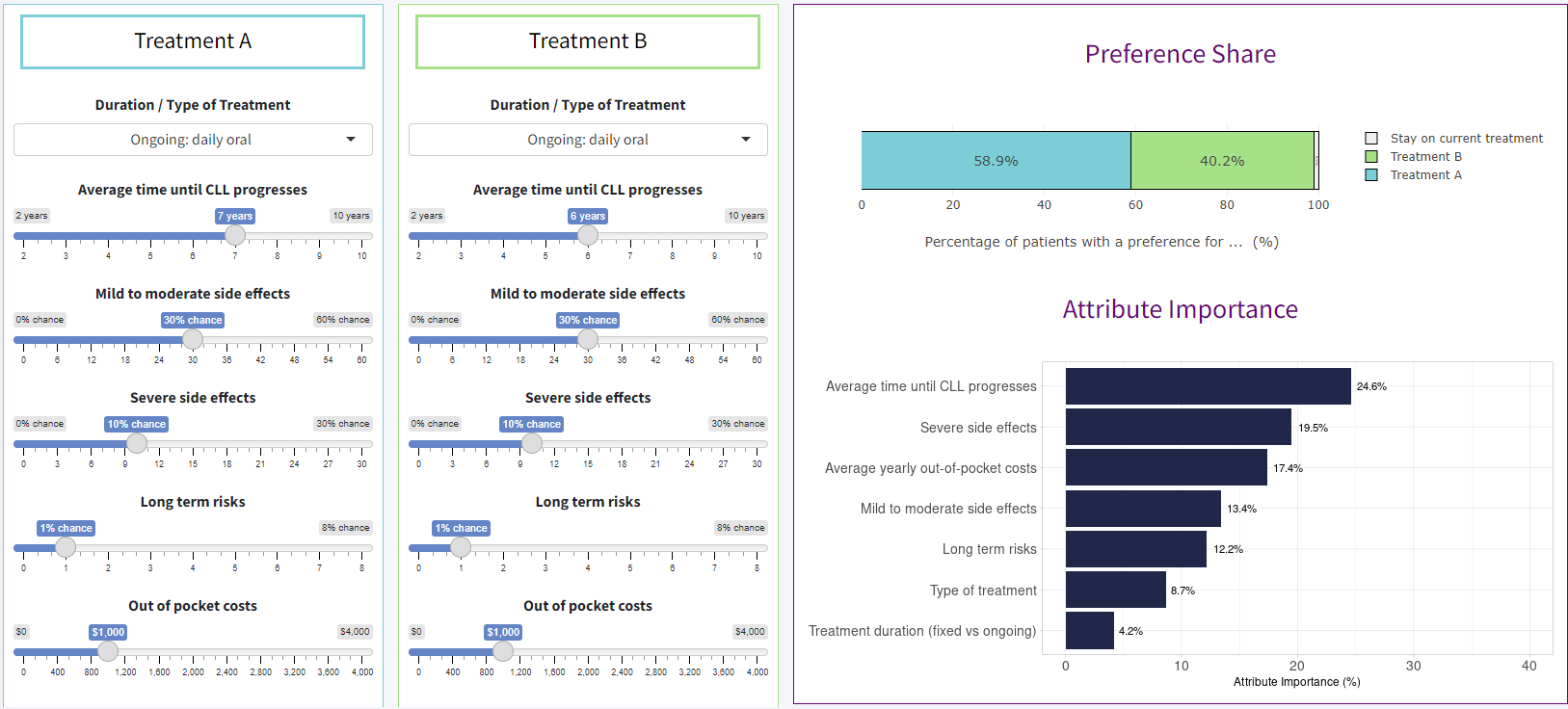


Figure S2 CLL Treatment preferences DCE Simulation 2

### Simulation 3: Treatment ‘risk’ trade off

In the third simulation we explored the potential impact of increasing the ‘*Likelihood of severe side effects*’ attribute level from 10% to 20%, holding everything else equal to the ongoing daily oral treatment from Simulation 1. As seen in Figure S3, preference share for the treatment with the lower chance of severe side effects (Treatment A) was 68.2% versus 30.3% for the treatment with greater chance of severe side effects (Treatment B).


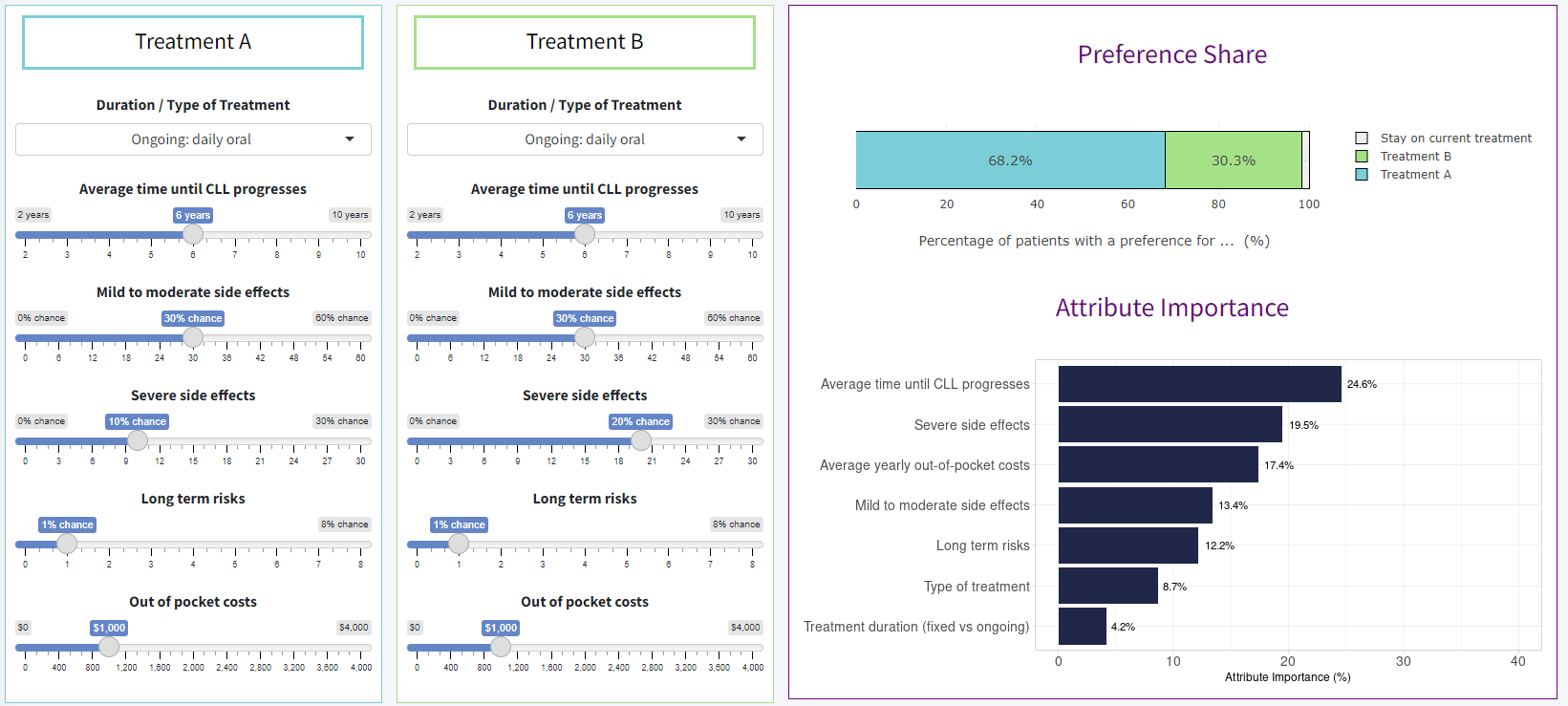


Figure S3 CLL Treatment preferences DCE Simulation 3

### Simulation 4: Out of pocket costs

Simulation 4 examined the impact on preference share of adjusting the ‘*Average annual out of pocket costs’* from $1000 to $2000. In this case, preference share for the treatment with the lower cost ($1000) is 62.3% versus 36.3% for the higher cost ($2000).

### Simulation 5: IV administration

This simulation compared an ‘ongoing daily oral alone’ therapy to a '24 months fixed' therapy (i.e., fixed daily oral for 24 months including IV for 6 months). In this case, preference share for the ‘ongoing daily oral alone’ therapy increased substantially (preference share: 62.7% versus ‘fixed daily oral plus IV’: 36%).
