# Supplementary material for: Understanding the experience, treatment preferences and goals of people living with chronic lymphocytic leukemia (CLL) in Australia
Source: BMC Cancer. 2024 Jul 11;24:831. doi: 10.1186/s12885-024-12589-9 (PMC11241996; doi:10.1186/s12885-024-12589-9)
Supplement: Supplementary file 2 — Supplementary Material 2 [file 12885_2024_12589_MOESM2_ESM.docx]

CLL Patient Experience: Patient Experience Index

| **Study Title:** | | **“Chronic Lymphocytic Leukaemia Patient Experience: PEI”** |
| --- | --- | --- |
| **Principal Investigator (Study Doctor):** | | Dr Simon Fifer |
| **Telephone:** | | +61 403 862 091 |
| **Local Legal Entity Name and Address:** | | Community and Patient Preference Research (CaPPRe): Level 20, 25 Bligh Street, Sydney Australia |
| **Study Sponsor** |  | CaPPRe |
| **Funder of Study** |  | Janssen ANZ |

Thank you for expressing interest in our research project! Your time and input is greatly appreciated.

The next few pages will include some questions that will help us determine your eligibility for the survey. Sometimes we have people trying to complete the survey who are not suitable, which will be picked up when we audit our data. In order to prevent this from occurring, we may need to ask you some additional questions, so we accurately capture the preferences of those with the condition.

If you are eligible, you will be taken to an Information Sheet and Consent Form. If you choose to participate, you will be taken directly to the main survey.

# Diagnosis Screener

## About you

### Device [SINGLE]

#### What type of device are you using to complete this survey?

##### Please select one answer

| **1** | Desktop computer |  |
| --- | --- | --- |
| **2** | Laptop computer |  |
| **3** | Standard tablet size |  |
| **4** | Mini tablet | ERROR |
| **5** | Mobile phone | ERROR |

IF A1:=4 or A1:=5 RAISE ERROR:

“Thank you so much for taking the time to click though to the survey. Unfortunately, the device you are using is too small – you won’t be able to see the whole survey. If you are able to complete the survey on a desktop computer, laptop or standard-sized tablet it’d be very much appreciated!

Please enter your email address and we will send you a direct link to the survey: OPEN TEXT BOX”

### FP_details [OPEN TEXT] – hidden for panels – SCRIPTING NOTE – Open link only question

#### Please enter your details below (please be assured that this information will only be used for the purpose of this research)

##### Please enter your response below

| **1** | First name | OPEN TEXT BOX |
| --- | --- | --- |
| **2** | Last name | OPEN TEXT BOX |
| **3** | Phone number | OPEN TEXT BOX |

A2:3 ERROR MESSAGE: “Please provide a valid phone number.”

### A3: Email [OPEN TEXT] – hidden for panels – SCRIPTING NOTE – Open link only question

#### Thank you for expressing interest in our research project. Please enter your email address below so that we can email you a personalised link to the survey (may be sent to your "junk" folder).

#### Please enter your email address below to receive a personalised link to the survey (may be sent to your "junk" folder). If at any point you need to take a break from the survey, you can stop and use the link sent to your email to continue from where you left off. *Please note we will not pass on your email or use it for any other purposes than for this study.*

##### Please enter a valid email address

|  | OPEN TEXT BOX | ERROR EMAIL INVALID |
| --- | --- | --- |

ERROR MESSAGE: “Please provide a valid email address.”

### A4: Country: Location [SINGLE]

#### Which country do you live in?

##### Please select one answer

| **1** | Australia |  |
| --- | --- | --- |
| **2** | Other (please specify) | SCREEN OUT |

### A5: Age [SINGLE]

#### Which category describes your age?

##### Please select one answer

| **1** | Less than 18 | SCREEN OUT |
| --- | --- | --- |
| **2** | 18-30 |  |
| **3** | 31-40 |  |
| **4** | 41-50 |  |
| **5** | 51-60 |  |
| **6** | 61-70 |  |
| **7** | 71-80 |  |
| **8** | 81 or older |  |
| **9** | Prefer not to answer |  |

IF 0=1 SCREEN OUT

### A6: Pharma [SINGLE]

#### Are you an employee of a pharmaceutical, medical device or vaccine company?

##### Please select one answer

| **1** | Yes | SCREEN OUT |
| --- | --- | --- |
| **2** | No |  |

### A7: Medicare [SINGLE]

#### Are you a citizen or permanent resident of the country that you are currently living in?

##### Please select one answer

| **1** | Yes |  |
| --- | --- | --- |
| **2** | No | SCREEN OUT |

### A8: Diagnosis [MULTI; RANDOM ORDER]

#### Have you been diagnosed with any of the following by a medical professional?

##### Please select all that apply

| **1** | Chronic Lymphocytic Leukaemia | REQUIRED |
| --- | --- | --- |
| **2** | Secusitic Leukaemia | SCREEN OUT |
| **3** | Follicular Lymphoma | FLAG FOR CHECKING |
| **4** | Lymphoblastic Lymphoma | FLAG FOR CHECKING |
| **5** | Melanoma | FLAG FOR CHECKING |
| **6** | Multiple Myeloma | FLAG FOR CHECKING |
| **7** | None of the above | SCREEN OUT |

SCREEN OUT IF A4:1 NOT SELECTED

### A9: Symptom check [OPEN TEXT]

#### Please describe your most severe symptoms of Chronic Lymphocytic Leukaemia (CLL)

##### Please enter your response

|  | OPEN TEXT BOX |  |
| --- | --- | --- |

Screen in text:

“We are pleased to inform you that you qualify to participate in this research project. Thank you for your time and interest! You will now be shown to the information sheet, consent form and survey. You are free to withdraw participation at any time by exiting the survey window.

If at any point you experience distress, you are advised to contact the healthcare professional that is helping you manage CLL or call one of the following hotlines:

Leukaemia Foundation Australia 1800 620 420 / Lymphoma Care Nurse Support Line: 1800 953 081

Screen out text:

“Thank you so much for taking the time to work through some of this questionnaire. Unfortunately, one of the responses you have given means you don’t qualify to take part.

If you are experiencing distress, you are advised to contact the healthcare professional that is helping you manage CLL or call one of the following hotlines:

Leukaemia Foundation Australia 1800 620 420 / Lymphoma Care Nurse Support Line: 1800 953 081

# Participation Information Form & Consent

**Protocol Number:** PCSONC00442, Version 3, 7th June, 2022: Page 1 of 5

| **Study Title:** | | **“Chronic Lymphocytic Leukaemia Patient Experience: PEI”** |
| --- | --- | --- |
| **Principal Investigator (Study Doctor):** | | Dr Simon Fifer |
| **Telephone:** | | +61 403 862 091 |
| **Local Legal Entity Name and Address:** | | Community and Patient Preference Research (CaPPRe): Level 20, 25 Bligh Street, Sydney Australia |
| **Study Sponsor** |  | CaPPRe |
| **Funder of Study** |  | Janssen ANZ |

## Introduction

This Participant Information Sheet and Consent Form tells you about the research project. It explains the purpose of the research, procedures and risks involved, the sort of information you will be asked about, how that information will be used and with whom it will be shared. Knowing what is involved will help you decide if you want to take part in the research. Please read this information carefully. Participation in this research is voluntary. If you don't wish to

**Protocol Number:** PCSONC00442, Version 3, 7^th^ June, 2022: Page 2 of 5

take part, you do not have to. If you choose not to participate, your treatment will not be affected. If you decide you want to take part in the research project, you will be asked for your consent to participate.

By consenting to participate you are telling us that you:

- Understand what you have read
- Consent to take part in the research project
- Consent to the use of your personal and health information as described

If you would like a copy of this participant information sheet and consent form to keep, you can print this screen.

##### Please click on each of the following headings to see the study details

**Purpose of the study**

Community and Patient Preference Research (CaPPRe) is working collaboratively with the pharmaceutical company, Janssen ANZ, who is funding this research on Chronic Lymphocytic Leukaemia (CLL).

CaPPRe are specialists in conducting research to determine people's preferences. We are looking at ways to gather the views from patients on what they think are the most important aspects of their experience living with CLL. In this research we are also hoping to understand what aspects of their experience could be improved and how.

**What’s involved?**

We would like to invite you to participate in a 30-40 minute online survey where you will be asked some questions about CLL. You will also be asked to complete a survey which asks you about the different areas of CLL healthcare; you will be asked to select which areas you are most and least satisfied with, and which areas are most and least important to you. We expect to recruit 30-40 participants in this study.

**Questions of a sensitive nature**

We have considered the views of patients when designing and wording the survey. It is possible that you may find some of the questions uncomfortable. Some questions in the surveys ask questions of a sensitive nature. If you feel uncomfortable you can stop at any time. Your participation is entirely voluntary.

If you find the survey stressful and would like to seek additional support and advice, please speak to your doctor or contact one of the following helplines:

- Leukaemia Foundation Australia 1800 620 420
- Lymphoma Care Nurse Support Line 1800 953 081

**Voluntary Participation/Right to Refuse or Withdraw**

Participation in any research project is voluntary. If you do not wish to take part, you do not have to. If you decide to take part and later change your mind, you are free to withdraw from the project at any stage. There is no formal process to withdraw consent, and you may withdraw consent at any point in time by closing the survey window. Your decision whether to take part or not to take part, or to take part and then withdraw, will not affect your routine treatment, or your relationship with those treating you. If you do withdraw your consent during the research project we will not collect additional personal information from you, although personal information already collected will be retained to ensure that the results of the research project can be measured properly and to comply with law. You should be aware that data collected up to the time you withdraw will form part of the research project results. If you have any questions about this or do not wish your initial data to be included in data analysis, please contact [rose.wilson@cappre.com.au](mailto:rose.wilson@cappre.com.au) or call 0467 952 347.

**Protocol Number:** PCSONC00442, Version 3, 7^th^ June, 2022: Page 3 of 5

**Confidentiality**

CaPPRe respects and understands your privacy is very important. By providing your consent you agree to us collecting and using personal and health information you have provided for the research project. Any information obtained in connection with this research project that can identify you will remain confidential. Your personal details will not be forwarded to any other parties, nor will you be contacted by CaPPRe for anything other than this research project unless you choose to be. Your data will be stored in a secure encrypted (protected) environment in Australian servers and only CaPPRe will have access to the data. There is no collection, transmission, analysis, storage and/or security of data outside of Australia at any time-point. Your information will only be used for the purpose of this research project and it will only be disclosed with your permission, except as required by law.

Any information you provide will be collected by CaPPRe. Any information that is shared will be done in report form. The personal information you provide, including sensitive personal information about you will not be shared.

It is anticipated that the results of this research project will be published and/or presented in a variety of forums. In any publication and/or presentation, information will be provided in a combined form such that no individual person can be identified. Janssen will be provided with a report of the findings containing only combined de-identified data. The research data will be kept securely for a period of 5 years.

**Payment** [HIDE FOR PANELS]

In appreciation for your time and participation, you will receive $75 as a e-gift card upon completion of the full survey (please note that your information will be collected for the purposes of sending this and will be deleted upon research completion). Alternatively, you can elect to have this reimbursement donated to a support group.

Sometimes we have people trying to complete the survey who do not have the condition, and this is picked up when we audit our data. If this occurs, we may need to ask you some additional questions, and of course reimbursement will only be paid to genuine patients. You will receive the e-gift card once the audit has been completed which will be approximately 2-3 weeks after your participation in the research study.

**Whom to contact about this study**

If you have any questions or concerns you can contact CaPPRe at [rose.wilson@cappre.com.au](mailto:rose.wilson@cappre.com.au) or call 0467 952 347.

**Protocol Number:** PCSONC00442, Version 3, 7^th^ June, 2022: Page 4 of 5

**Reportable Adverse Events Including Drug Side Effects / Product Quality Issues**

If during the survey, you report a side effect or product quality issue that you have experienced while taking a medicine from the pharmaceutical company funding this research, there is an obligation by the pharmaceutical company to report this side effect (or Adverse Event) or product quality issue as part of the ongoing monitoring of their product. This is a mandatory requirement by the pharmaceutical company regarding its own products and therefore, any information you provide during the survey relating to their specific side effects will need to be sent to the pharmaceutical company for their records. In this instance, you will be asked whether or not you are willing to waive the confidentiality given to you under the Market Research Codes of conduct specifically in relation to that adverse event/product quality issue. Everything else you say during the course of the survey will continue to remain confidential, and you will still have the option to remain anonymous if you so wish.

Please note that we are only reporting side effects of products from the pharmaceutical company funding this research. If you have concerns regarding side effects with any product you have received for CLL, we recommend that you seek advice from your GP or specialist.

### AE [SINGLE]

#### Please indicate what information you permit us to share with the sponsoring company’s Drug Safety Department should you mention an Adverse Event or Product Quality Complaint associated with their specific products.

##### Please select one answer

| **1** | **Yes**, and I give permission to be **recontacted** by the Drug Safety Department for more information.  **Contact information** (name, email address, phone number) **will** be shared  De-identified information (gender, age group, etc) will also be shared. | **Hidden for panels** |
| --- | --- | --- |
| **2** | **Yes**, but I do not wish to be recontacted.  **Contact information** (name, email address, phone number) **will not** be shared  De-identified information (gender, age group, etc) will be shared. |  |
| **3** | **I do not give permission** for any adverse events to be reported and understand **I will not be able to participate in this research.** | SCREEN OUT |

**Protocol Number:** PCSONC00442, Version 3, 7^th^ June, 2022: Page 5 of 5

## Consent

I hereby consent to my involvement in this research project.

I acknowledge that the nature, purpose and risks of the research project and alternatives to participation have been fully explained to my satisfaction by CaPPRe personnel. Specifically, the details of the research proposed and the anticipated length of time it will take have been explained to me.

- I am 18 years of age or over
- I freely agree to participate in this research project per the conditions in the Participant Information Sheet.
- I understand that my involvement in this research may not be of any direct benefit to me.
- I have been given the opportunity to discuss the project with a member of my family or another person.
- I have been told that neither my personal information nor information regarding my medical history will be divulged to unauthorised third parties and the findings from this research will not include any identifying information in publication or other forms of dissemination.
- I understand that I am free to withdraw from the research at any stage. If I decide to withdraw from the research, I agree that the information collected about me up to the point when I withdraw may continue to be processed.
- I do not have a prior or current relationship with CaPPRe.
- I declare that all my questions have been answered to my satisfaction.
- I understand that the results may be used for commercial purposes.
- I understand that I may be contacted by email to clarify my survey responses and/or provide additional information about my current treatment.

### Consent [SINGLE]

#### I have read, or have had read to me, and I understand the Participant Information Sheet.

##### Please select one answer

| **1** | I consent voluntarily to take part in this research |  |
| --- | --- | --- |
| **2** | I do not consent to be a part of this research | SCREEN OUT |

##### Please remember to print screens related to the participant information, your consent to AE reporting and your consent to participate in this research if you would like to keep a record of this information.

Please keep in mind that if at any point you need to take a break from the survey, you can stop and use the link sent to your email to continue from where you left off. It is acceptable and appropriate for a primary carer to be involved in assisting you complete the survey if required (i.e. to assist with the on-line component and explanations of survey requirements). Should you have any questions, please contact [rose.wilson@cappre.com.au](mailto:rose.wilson@cappre.com.au) or call 0467 952 347.

# Disease & Treatment Background

## Questions about living with CLL

### Time symptoms [GRID]

#### When do you first recall experiencing symptoms of CLL?

##### Please select the year and month

| **1** | Year | LIST 1950+ |
| --- | --- | --- |
| **2** | Month | LIST 1-12 |
| **3** | Don’t know |  |

### Time diagnosed [GRID]

#### When were you first diagnosed with CLL by a healthcare professional?

##### Please select the year and month

| **1** | Year | LIST 1950+ |
| --- | --- | --- |
| **2** | Month | LIST 1-12 |
| **3** | Don’t know |  |

### Healthcare professional [MULTI; RANDOM ORDER except code 5]

#### Which of the following healthcare professionals help you manage your CLL?

##### Please select all that apply

| **1** | Haematologist |  |
| --- | --- | --- |
| **2** | Oncologist |  |
| **3** | General practitioner / my local doctor |  |
| **4** | Nurse / nurse practitioner / nurse consultant / registered nurse |  |
| **5** | Other (please specify) | KEEP POSITION |

FLAG IF C3:1 or 2 or 3 NOT SELECTED

### Setting [SINGLE]In what setting do you mainly receive healthcare treatment for CLL? I.e., if you have consultations with physicians, are these through a public or private provider? Are any hospital stays or out-patient visits in public or private hospitals?

**(If this has changed over time, or you use a mix of public/private providers please select ‘a combination of public/private’)?**

##### Please select your answer

| **1** | Public |  |
| --- | --- | --- |
| **2** | Private |  |
| **3** | A combination of Public and Private |  |
| **4** | Other (please specify) |  |
| **5** | Don’t know / unsure |  |

# Best-Worst Scale task

## In this section of the survey, we would like to understand your personal experience with aspects of the healthcare pathway for CLL

On the next 11 screens, you will be shown a number of scenarios, with each set showing 6 different items.

Each item represents an aspect of the healthcare system for the treatment of CLL. Some of the screens may seem similar but there are small differences between them.

- Please select which aspect you are **MOST SATISFIED** and which aspect you are **LEAST SATISFIED** with personally.
- Please also select which aspect is **MOST IMPORTANT** and which aspect is **LEAST IMPORTANT** to you personally.

If you are struggling to choose a statement for one of the columns, please try to select the statement that is most appropriate. For example, if you are satisfied with all 6 statements shown and you are struggling to choose one for the 'least satisfied' column, please select the one that is the least satisfactory out of the 6 shown. You will be given the chance to justify your answers at the end of the task.

BWS ATTRIBUTE LIST: Insert according to grid

|  | **DOMAIN** | Description (Hover-over text) |
| --- | --- | --- |
| **1** | **Time to diagnosis** | - The length of time from developing symptoms through to being diagnosed – whatever this looked like for you. |
| **2** | **The quality of information available about your condition and care** | - Having clear, concise, relevant information in a format that works for you (e.g., provided to you by your healthcare team/online/Apps/podcasts). |
| **3** | **Your involvement in decision making** | - How involved you are in decisions about your treatment and care, e.g., when selecting specific medication and/or when developing a treatment plan |
| **4** | **The quality of your healthcare team – access to your key healthcare professional/s, consistency of care, and their communication with you and between each other** | - Suitable access to your key healthcare professional (e.g., haematologist), at regular intervals that you feel are most beneficial to you or in acute situations where urgent access is required. - Being able to see the same trusted healthcare professional/s on-going for your treatment and care. - How well your needs are met in any interactions with your healthcare team (including doctors, nurses, care coordinators). - The extent to which different members of your healthcare team (e.g., haematologist/GP/clinical nurse specialist) communicate with each other about your condition and care. They may be healthcare professionals within the same service or in different services. |
| **5** | **Treatment logistics** | - The broad impact that following a treatment and care plan has on you., i.e., day-to-day difficulties of arranging and attending treatment sessions. |
| **6** | **Access to, and effectiveness of, medication** | - Your access to medication for your condition. - How effective the medication prescribed by your healthcare professional/s is in treating your condition. |
| **7** | **Side effects of medication** | - Side-effects you may experience from medication prescribed by your healthcare professional/s. |
| **8** | **Monitor & identify progress/deterioration** | - The ability to monitor day-to-day and long-term changes in your physical and overall wellbeing, for yourself, and by your healthcare professionals (e.g., pain, fatigue) and adjustments to treatment and care based on this. |
| **9** | **Access to other treatments/services (including a care coordinator), to support physical health, mental health, overall wellbeing (holistic approach)** | - Other services could include seeing a psychologist or exercise physiologist. Complementary treatments could include acupuncture, massage, mind-body techniques, and management strategies for increased wellness (e.g., access to dietitians/physiotherapists/occupational therapists/psychologists). - Having someone who is assigned to you (e.g., social worker or peer support worker) to help you navigate the healthcare system and offer emotional support and guidance. |
| **10** | **Support for your 'support person'** | - Information/websites specifically for significant others (e.g., spouse, partner, friend etc) and support groups where family members/friends can talk with others in similar situations. |
| **11** | **CLL-related costs** | - The overall impact that having CLL has on your financial wellbeing, e.g., how much you are out-of-pocket, and the impact of loss of income. |

Please consider your personal experiences with each of these aspects of the healthcare pathway throughout the treatment and management of your CLL.

There are no right or wrong answers. We are purely interested in which aspects of the healthcare pathway are important and satisfactory to you.


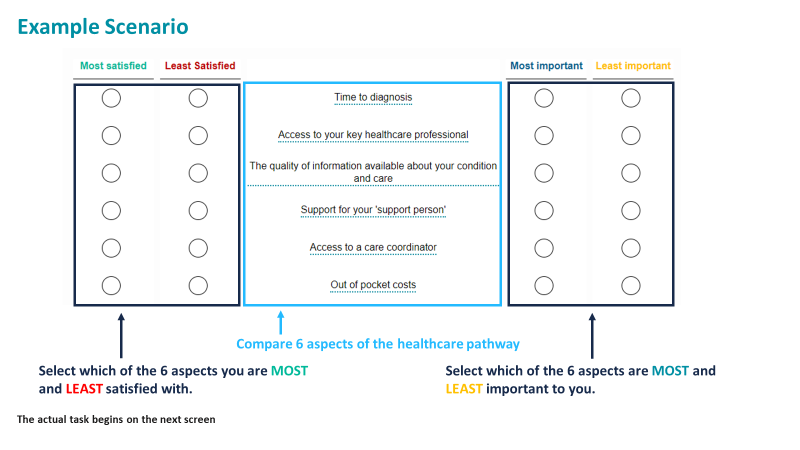


INSERT FOR EACH SCENARIO:

### Scenario 1 [BWS TASK]

#### Thinking about your personal experiences with the healthcare pathway throughout the treatment and management of your CLL:

- Please select which of the following aspect you are **MOST SATISFIED** and which aspect you are **LEAST SATISFIED** with personally.
- Please also select which aspect is **MOST IMPORTANT** and which aspect is **LEAST IMPORTANT** to you personally.

#### You can use your mouse to hover over the statements to see more information

##### Please select one answer per column

| **MOST IMPORTANT** | **LEAST IMPORTANT** | INSERT ACCORDING TO BWS DESIGN | **MOST SATISFIED** | **LEAST SATISFIED** |
| --- | --- | --- | --- | --- |
|  |  |  |  |  |
|  |  |  |  |  |
|  |  |  |  |  |
|  |  |  |  |  |
|  |  |  |  |  |
|  |  |  |  |  |
|  |  |  |  |  |

There are no right or wrong answers. You will be given an opportunity to explain your choices later in the survey.

# Rescaling

Shown below are the aspects you nominated as **MOST** and **LEAST important to you.**

When more than one aspect was nominated with equal importance, we will show all aspects with equal importance.

**We'd like to get a better idea about how important these aspects are to you on a scale from 0 (not important at all) to 10 (extremely important).**

### MOST important [SINGLE]

##### Please rate how important each of the following aspects are to you for the healthcare pathway for CLL on a scale from 0 (“Not important at all”) to 10 (“Extremely important”)

##### Please select one answer

| INSERT ACCORDING TO BWS DESIGN | 1-10 SCALE |
| --- | --- |

### LEAST important [SINGLE]

##### Please rate how important each of the following aspects are to you for the healthcare pathway for CLL on a scale from 0 (“Not important at all”) to 10 (“Extremely important”)

##### Please select one answer

| INSERT ACCORDING TO BWS DESIGN | 1-10 SCALE |
| --- | --- |

Shown below are the aspects you nominated as MOST and LEAST satisfied with.

When more than one aspect was nominated with equal satisfaction, we will show all aspects with equal satisfaction.

We'd like to get a better idea about how satisfied you are with these aspects on a scale from 0 (not satisfied at all) to 10 (completely satisfied).

### MOST satisfied [SINGLE]

##### Please rate how satisfied you are with each of the following aspects across the healthcare pathway for CLL on a scale from 0 (“Not at all satisfied”) to 10 (“Extremely satisfied”)

##### Please select one answer

| INSERT ACCORDING TO BWS DESIGN | 1-10 SCALE |
| --- | --- |

### LEAST satisfied [SINGLE]

##### Please rate how satisfied you are with each of the following aspects across the healthcare pathway for CLL on a scale from 0 (“Not at all satisfied”) to 10 (“Extremely satisfied”)

##### Please select one answer

| INSERT ACCORDING TO BWS DESIGN | 1-10 SCALE |
| --- | --- |

# Preference feedback

## In the next section we’d like to get your feedback about the scenarios you have just completed

### Understanding [SINGLE]

##### Please rate your understanding of the scenarios you have just completed on a scale from 1 ("Did not understand the scenarios at all") to 10 ("Completely understood the scenarios"). Please select one answer

| 1-10 SCALE |
| --- |

### Ease [SINGLE]

##### How easy did you find answering these scenarios on a scale from 1 ("Very Difficult") to 10 ("Very Easy")? Please select one answer

| 1-10 SCALE |
| --- |

### Feedback attributes [GRID]

#### Please select whether you understood the definitions for each aspect of the healthcare system, or if they were not clear.

##### Please select an answer for each.

|  |  | **I understood this definition** | **This definition was not clear to me** |
| --- | --- | --- | --- |
|  | INSERT ACCORDING TO BWS DESIGN | Yes/no | Yes/no |
|  |  | Yes/no | Yes/no |
|  |  | Yes/no | Yes/no |
|  |  | Yes/no | Yes/no |
|  |  | Yes/no | Yes/no |
|  |  | Yes/no | Yes/no |
|  |  | Yes/no | Yes/no |
|  |  | Yes/no | Yes/no |

### Process [OPEN TEXT]

##### Please describe your thought process while completing these scenarios. What were you thinking about and focusing on while completing the scenarios? Please enter your response

| OPEN TEXT BOX |
| --- |

# Least Satisfied Domains

## In the next section we would like to get your feedback about the 4 aspects of the healthcare pathway that you are least satisfied with

### Dissatisfaction why [OPEN TEXT]

##### Based on the choice tasks in the last section of the survey, the following 4 aspects of the healthcare pathway are the ones that are most important to you, but that you are least satisfied with:

SHOW 4 LEAST SATISFIED + MOST IMPORTANT DOMAINS

##### Please explain your reasons for each. Please enter your response for each

| **1** | PIPE: Satisfaction domain rank 15 | OPEN TEXT |
| --- | --- | --- |
| **2** | PIPE: Satisfaction domain rank 14 | OPEN TEXT |
| **3** | PIPE: Satisfaction domain rank 13 | OPEN TEXT |
| **4** | PIPE: Satisfaction domain rank 12 | OPEN TEXT |

### Dissatisfaction probed [MULTI GRID]

MATCH PROBES FROM GRID FOR EACH DOMAIN TO THE 4 LEAST SATISFIED + MOST IMPORTANT DOMAINS

##### a) Please select all of the areas below that you believe contribute to your dissatisfaction for each aspect of treatment and healthcare

##### b) Please describe in more detail the reason for your dissatisfaction

##### c) Please describe your recommendation for improving this area of the healthcare pathway

##### Please select all that apply and enter your response for each

SHOW DOMAIN RANK 11

|  | **DOMAIN** | a) | b) | c) |
| --- | --- | --- | --- | --- |
|  |  | RADIO BTN | OPEN TEXT | OPEN TEXT |

**EXAMPLE IF QUALITY OF INFORMATION WAS RANKED 15 FOR SATISFACTION**

|  | **Time to diagnosis** |  |  |  |
| --- | --- | --- | --- | --- |
| **1** | I am not satisfied with the speed of referral for diagnostic testing, and/or to a specialist, e.g., haematologist |  |  |  |
| **2** | I am not satisfied with my understanding of the diagnostic pathway, e.g., the purpose of each test, order of diagnostic tests/the plan for diagnostic testing, in order to reach a diagnosis and/or eliminate other conditions |  |  |  |
| **3** | I am not satisfied with the speed of diagnostic testing, e.g., diagnostic tests were run one after the other, instead of at the same time |  |  |  |
| **4** | I am not satisfied with my awareness and understanding of CLL before diagnosis |  |  |  |
| **5** | Other [I am not satisfied with something else relating to the time to diagnosis (please specify)] |  |  |  |

REPEAT FOR DOMAINS RANKED 14,13,12 & 11 CHOOSING FROM LIST OF PROBES PER DOMAIN BELOW

|  | **The quality of information available about your condition and care** |  |  |  |
| --- | --- | --- | --- | --- |
| **1** | I am not satisfied with the guidance I have received on how to navigate resources available to find information that is relevant to my situation |  |  |  |
| **2** | I am not satisfied with the information/knowledge available in public spaces about CLL (e.g., pharmacies, media etc) |  |  |  |
| **3** | I am not satisfied with the information/knowledge about CLL provided by my health care professional |  |  |  |
| **4** | I am not satisfied with the format in which information has been delivered to me (e.g., booklets, apps, online, podcasts) |  |  |  |
| **5** | I am not satisfied with the level of trust and confidence I have in the information on my condition |  |  |  |
| **6** | I am not satisfied with the way the information is written, i.e., it is not easy for me to understand (especially if English is not your first language) |  |  |  |
| **7** | I am not satisfied with the provision of information because there is too much, maybe making it overwhelming and/or make it difficult to work out what is relevant to me |  |  |  |
| **8** | Other [I am not satisfied with something else relating to the quality of information available about my condition and care (please specify)] |  |  |  |

|  | **Your involvement in decision making** |  |  |  |
| --- | --- | --- | --- | --- |
| **1** | I am not satisfied with my level of inclusion in decisions around medication |  |  |  |
| **2** | I am not satisfied with my level of inclusion in developing a treatment plan alongside medication, e.g., diet and exercise |  |  |  |
| **3** | I am not satisfied with the extent to which healthcare professional/s ask what is important to me, when making treatment and healthcare plans |  |  |  |
| **4** | I am not satisfied with how my healthcare team set and work towards achieving aligned goals |  |  |  |
| **5** | I am not satisfied with my understanding of the treatment options available and what to expect when undergoing treatments |  |  |  |
| **6** | Other [I am not satisfied with something else relating to my involvement in decision making (please specify)] |  |  |  |

|  | **The quality of your healthcare team – access to your key healthcare professional/s, consistency of care, and their communication with you and between each other** |  |  |  |
| --- | --- | --- | --- | --- |
| **1** | I am not satisfied with my consistency of care, i.e., being able to see the same healthcare professionals on-going for treatment and care |  |  |  |
| **2** | I am not satisfied with my ability to contact CLL healthcare professional/s who can help me as required, i.e., obtaining advice or care at short notice in more urgent situations or after hours such as evenings or weekends |  |  |  |
| **3** | I am not satisfied with the ongoing availability of reoccurring consultations with key healthcare professionals |  |  |  |
| **4** | I am not satisfied with the fact I am not able to see my healthcare professional/s face-to-face (i.e., appointments now telehealth due to covid) |  |  |  |
| **5** | I am not satisfied with how often healthcare professionals check-in with me to see if I am happy with my care and treatment plans |  |  |  |
| **6** | I am not satisfied with how much my healthcare team listen to me and understand what I am going through |  |  |  |
| **7** | I am not satisfied with how much my healthcare team empathise and provide me with hope regarding CLL |  |  |  |
| **8** | I am not satisfied with my capacity to find the right healthcare professional - someone I feel comfortable with and who understands my needs |  |  |  |
| **9** | I am not satisfied with the quality of information my healthcare team provides and discusses with me |  |  |  |
| **10** | I am not satisfied with the quality of any communications outside of consultations e.g., emails/telephone calls |  |  |  |
| **11** | I am not satisfied with how much I am understood and treated holistically, i.e., healthcare professionals understanding of my social and mental health needs as well as physical, and how these are addressed and treated |  |  |  |
| **12** | I am not satisfied with the extent to which different members of my healthcare team (e.g., haematologist, other specialists, pharmacists, care co-ordinators, nurses) communicate with each other about my condition and care. They may be healthcare professionals within the same service or in different services. |  |  |  |
| **13** | I am not satisfied with healthcare professional access to and use of ‘My HealthRecord’ (an online summary of your key health information) |  |  |  |
| **14** | Other [I am not satisfied with something else relating to the quality of your healthcare team (please specify)] |  |  |  |

|  | **Treatment logistics** |  |  |  |
| --- | --- | --- | --- | --- |
| **1** | I am not satisfied with the logistics of getting to my healthcare appointment (treatment/consultations), due to distance to the hospital/healthcare centre or it being in a difficult location. |  |  |  |
| **2** | I am not satisfied with lost time due to treatment obligations |  |  |  |
| **3** | I am not satisfied with my reduced capacity to work, due to treatment |  |  |  |
| **4** | I am not satisfied with the burden (i.e., discomfort) associated with receiving medication (e.g., tenderness/infusion site pain) |  |  |  |
| **5** | I am not satisfied with the availability of treatment administered in the home (i.e., hospital in the home) |  |  |  |
| **6** | Other [I am not satisfied with something else relating to treatment logistics (please specify)] |  |  |  |

|  | **Access to, and effectiveness of, medication** |  |  |  |
| --- | --- | --- | --- | --- |
| **1** | I am not satisfied with my level of access to new medication/s |  |  |  |
| **2** | I am not satisfied with my level of access to clinical trials |  |  |  |
| **3** | I am not satisfied with the number of times my healthcare team [i.e. treating physician(s)] have changed my medication for CLL |  |  |  |
| **4** | I am not satisfied with how well medication/s works to reduce or prevent further symptoms associated with CLL (e.g., pain/fatigue) |  |  |  |
| **5** | I am not satisfied with how well medication works to prolong progression free survival i.e., how well medication works to stop the CLL from progressing |  |  |  |
| **6** | Other [I am not satisfied with something else relating to access and effectiveness of medication (please specify)] |  |  |  |

|  | **Side effects of medication** |  |  |  |
| --- | --- | --- | --- | --- |
| **1** | I am not satisfied with my level of preparedness for side effects ([i.e., the amount of information provided to me by my healthcare professionals on specific side effects (e.g. diarrhoea, nausea, fatigue, headache, musculoskeletal pain, bruising, rash, increased blood pressure)] |  |  |  |
| **2** | I am not satisfied with the support I was given for side effect management (e.g., advice from healthcare professionals) |  |  |  |
| **3** | I am not satisfied with the impact of side-effects from medication on my day-to-day life |  |  |  |
| **4** | I am not satisfied with the long-term side effects of treatment; e.g., effects on bone marrow, risk of infection |  |  |  |
| **5** | Other [I am not satisfied with something else relating to side effects of medication (please specify)] |  |  |  |

|  | **Monitor & identify progress/deterioration (and adjustments to treatment and care based on this)** |  |  |  |
| --- | --- | --- | --- | --- |
| **1** | I am not satisfied with my ability to identify and track changes (both progression and deterioration) in physical health and overall wellbeing |  |  |  |
| **2** | I am not satisfied with the availability of tools to help me track changes in my physical and/or mental health |  |  |  |
| **3** | I am not satisfied with my healthcare professional's ability to identify and track changes (both progression and deterioration) in physical health and overall wellbeing |  |  |  |
| **4** | Other [I am not satisfied with something else relating to monitoring & identifying progress/deterioration (please specify)] |  |  |  |

|  | **Access to other treatments/services including a care coordinator - to support physical health, mental health, overall wellbeing (holistic approach)** |  |  |  |
| --- | --- | --- | --- | --- |
| **1** | I am not satisfied with the level of support I get for help with lifestyle changes, e.g., diet, exercise, sleep |  |  |  |
| **2** | I am not satisfied with the availability of appointments for complementary therapies/treatments, e.g., massage/acupuncture/mental health support |  |  |  |
| **3** | I am not satisfied with the fact I miss out on potential benefits of complementary therapies/treatments e.g., to reduce symptoms and side-effects of CLL and treatment |  |  |  |
| **4** | I am not satisfied with my level of access to support groups that provide holistic support for CLL |  |  |  |
| **5** | I am not satisfied with my access to support for life planning and/or financial management, e.g., how to manage broader aspects of life following impact of diagnosis |  |  |  |
| **6** | I am not satisfied with my access to a care coordinator |  |  |  |
| **7** | Other [I am not satisfied with something else relating to access to other treatments/services including a care coordinator (please specify)] |  |  |  |

|  | **Support for your 'support person'** |  |  |  |
| --- | --- | --- | --- | --- |
| **1** | I am not satisfied with the educational information available that is designed to support the person who supports me |  |  |  |
| **2** | I am not satisfied with the emotional support available for the person who supports me (e.g., peer support, counselling) |  |  |  |
| **3** | I am not satisfied with the financial support available for the person who supports me |  |  |  |
| **4** | I am not satisfied with my carer/s level of understanding about the depth of my condition |  |  |  |
| **5** | I am not satisfied with the availability of respite services for the person who supports me, i.e., services which provide a break in caring duties |  |  |  |
| **6** | I am not satisfied with the level of assistance provided for my support person to help them understand how to navigate the system |  |  |  |
| **7** | I am not satisfied with my support persons level of inclusion in treatment and care planning, e.g., attending specialist/specialist appointments |  |  |  |
| **8** | Other [I am not satisfied with something else relating to the support available for my support person (please specify)] |  |  |  |

|  | **CLL related costs** |  |  |  |
| --- | --- | --- | --- | --- |
| **1** | I am not satisfied with the out-of-pocket cost for my medication |  |  |  |
| **2** | I am not satisfied with the out-of-pocket cost transport and/or parking |  |  |  |
| **3** | I am not satisfied with the out-of-pocket cost complementary treatments |  |  |  |
| **4** | I am not satisfied with the out-of-pocket cost for specialist-gap payments |  |  |  |
| **5** | I am not satisfied with the financial implications of a reduced capacity for work (for you and/or your partner) |  |  |  |
| **6** | Other [I am not satisfied with the out-of-pocket cost for something else relating to my management and treatment for CLL (please specify)] |  |  |  |

### Other feedback [OPEN TEXT]

#### Do you have any other comments or suggestions for improving or optimising the CLL healthcare pathway?

##### Please enter your response

OPEN TEXT BOX

|  |
| --- |

# Treatment

## Final treatment questions

### CLL treatment / stage [SINGLE]

#### What is your current treatment status for CLL? Some options below refer to CLL treatment as ‘lines of treatment’. Please refer to the definition provided below.

A line of treatment refers to a set treatment approach which can include one drug or a combination of drugs that are taken until a patient stops that treatment or switches to another. E.g. 1st-line treatment refers to the initial treatment regime received. 2nd-line treatment refers to the second treatment regime received etc.

##### Please select one answer

| **1** | I have been diagnosed with CLL, however have not yet received any treatment | GO TO H3 |
| --- | --- | --- |
| **2** | I am currently on 1^st^-line treatment |  |
| **3** | I am not currently on treatment due to achieving remission / minimal disease activity from 1^st^-line treatment |  |
| **4** | I am currently on 2^nd^-line treatment |  |
| **5** | I am not currently on treatment due to achieving remission / minimal disease activity from 2^nd^-line treatment |  |
| **6** | I am currently on 3^rd^ or 4^th^ -line treatment |  |
| **7** | I am not currently on treatment due to achieving remission / minimal disease activity from 3^rd^ or 4^th^-line treatment |  |
| **8** | Other (please specify) | OPEN TEXT |
| **9** | Don’t know |  |

### Treatment regime received (MULTI)

#### Please indicate which of the following treatment regimens / approaches you have received for CLL? Please select both options if you have received both regimens / approaches.

#### Please refer to some examples of Chemoimmunotherapy and Targeted Therapy below:

##### Chemoimmunotherapy: Fludarabine + Cyclophosphamide + Rituximab (FCR); Bendamustine + Rituximab; Chlorambucil + Obinutuzumab)

##### Targeted Therapy: Ibrutinib, Venetoclax, Acalabrutinib

##### Please select all that apply

| **1** | Chemoimmunotherapy |  |
| --- | --- | --- |
| **2** | Targeted therapy |  |

### CLL severity [SINGLE]

#### How would you describe the impact of your CLL on your quality of life?

##### Please select one answer

| **1** | **No limitation** to my quality of life |  |
| --- | --- | --- |
| **2** | **Mild: Minimal impact** to quality of life. |  |
| **3** | **Moderate: Marked impact** to quality of life.  Affects ability to perform daily tasks of living and physical function. May impact mental well-being. |  |
| **4** | **Severe: Great impact** to quality of life.  Cannot perform major daily tasks of living without pain or dysfunction. Large impact to physical and mental well-being |  |
| **5** | Other (please specify) | OPEN TEXT |
| **6** | Don’t know |  |

### Treatment experience [MULTI] SHOW IF EVER RECEIVED TREATMENT IE H1: 2-8:1 or 2

#### Which of the following kinds of medications have you ever taken for CLL?

##### Please select all that apply

| **1** | Oral (swallowed by mouth in pill or tablet form) |  |
| --- | --- | --- |
| **2** | Injection under the skin |  |
| **3** | Intravenous infusion |  |
| **4** | Other (please specify) | OPEN TEXT BOX |
| **5** | Don’t know/unsure |  |

### Treatment costs [NUMERIC GRID]

#### Roughly, how much do you spend on out-of-pocket costs associated with treatment, per year, for each of the following:

##### Please enter your response for each

| **1** | Visits to healthcare professionals | NUMERIC TEXT BOX |
| --- | --- | --- |
| **2** | Cost of medication | NUMERIC TEXT BOX |
| **3** | Cost of complementary treatments e.g., complementary medicine, lifestyle changes | NUMERIC TEXT BOX |
| **4** | Travel costs associated with treatments | NUMERIC TEXT BOX |
| **5** | Private health insurance | NUMERIC TEXT BOX |
| **6** | Other (please specify) | NUMERIC TEXT BOX |
|  | TOTAL | SUM |

ERROR MESSAGE: “Please enter a number”

### Caregiver [SINGLE]

#### Do you have a key person who supports you with your management of CLL? This could be a partner, family member or friend.

##### Please select one answer

| **1** | Yes |  |
| --- | --- | --- |
| **2** | No |  |
| **3** | Don’t know / unsure |  |

### Services experience [MULTI]

#### Please select all of the services that you have been involved with or had experience with for the management of your CLL:

##### Please select all that apply

| **1** | National Disability Insurance Scheme (NDIS) involvement |  |
| --- | --- | --- |
| **2** | Patient support organisation (e.g. Leukaemia Foundation, Lymphoma Australia) |  |
| **3** | Pharmaceutical Company Patient Support Program |  |
| **4** | Respite centre |  |
| **D5** | “Safe café” or similar (support from healthcare professional in safe, non-medical setting) |  |
| **6** | Medication delivery system |  |
| **7** | Treatment, prescription, or appointment reminders |  |
| **8** | Patient advocacy groups, i.e., patient charities/support groups |  |
| **9** | Care coordinator (e.g., social worker or peer support worker) |  |
| **10** | Other (please specify) |  |
| **11** | Don’t know / unsure |  |
| **12** | None of the above |  |

### Involvement in treatment decision [RANK]

#### What is your preference when it comes to your involvement in treatment decisions e.g., when selecting a new CLL medication?

##### Please rank the options below by drag and drop from 1 to 5 where 1 is your most preferred option and 5 is your least preferred option

| **1** | I prefer to make the decision about which treatment I will receive |  |
| --- | --- | --- |
| **2** | I prefer to make the final decision about my treatment after seriously considering my doctor’s opinion |  |
| **3** | I prefer that my doctor and I share the responsibility for deciding which treatment is best for me |  |
| **4** | I prefer that my doctor make the final decision about which treatment will be used but seriously consider my opinion |  |
| **5** | I prefer to leave all decisions regarding treatment to my doctor |  |

### Role of Pharmaceuticals [OPEN TEXT]

#### What role do you expect pharmaceutical companies (the companies that make medications) to have in your healthcare?

##### Please enter your response

OPEN TEXT BOX

|  |
| --- |

#### Is there anything additional they could/should be doing to your healthcare experience?

##### Please enter your response

OPEN TEXT BOX

|  |
| --- |

# Demographics

## Final questions about you

### Gender [SINGLE]

#### Which of the following best describes your gender identity?

##### Please select one answer

| **1** | Male |  |
| --- | --- | --- |
| **2** | Female |  |
| **3** | Non-binary/gender fluid |  |
| **4** | Prefer to self-describe (please specify) | OTHER |
| **5** | Prefer not to answer |  |

### Occupation [SINGLE]

#### What is your occupation status?

##### Please select one answer

| **1** | Working (full-time) |  |
| --- | --- | --- |
| **2** | Working (part-time) |  |
| **3** | Working (casual) |  |
| **4** | Student |  |
| **5** | Not working |  |
| **6** | Home duties and/or caring responsibilities |  |
| **7** | Retired |  |
| **8** | Other (please specify) | OTHER |
| **9** | Prefer not to answer |  |

### Household [SINGLE]

#### Which of the following best describes your household?

##### Please select one answer

| **1** | Couple with no children |  |
| --- | --- | --- |
| **2** | Couple family with children |  |
| **3** | One parent family |  |
| **4** | Single person household |  |
| **5** | Group household (i.e., shared) |  |
| **6** | Other (please specify) | OTHER |
| **7** | Prefer not to answer |  |

### State [SINGLE]

#### Which state/territory do you currently live in?

##### Please select one answer

| **1** | ACT |  |
| --- | --- | --- |
| **2** | NSW |  |
| **3** | VIC |  |
| **4** | QLD |  |
| **5** | SA |  |
| **6** | WA |  |
| **7** | TAS |  |
| **8** | NT |  |

### Area [SINGLE]

#### How would you best describe the area you live in?

##### Please select one answer

| **1** | Metro/city |  |
| --- | --- | --- |
| **2** | Regional |  |
| **3** | Rural |  |

### Income [SINGLE]

#### Which of the following categories best describes your household’s annual gross income (before tax)?

##### Please select one answer

| **1** | Nil income |  |
| --- | --- | --- |
| **2** | $1-$7,799 (i.e., $1-$149 a week) |  |
| **3** | $7,800-$15,599 (i.e., $150-$299 a week) |  |
| **4** | $15,600-$20,799 (i.e., $300-$399 a week) |  |
| **5** | $20,800-$25,999 (i.e., $400-$499 a week) |  |
| **6** | $26,000-$33,799 (i.e., $500-$649 a week) |  |
| **7** | $33,800-$41,599 (i.e., $650-$799 a week) |  |
| **8** | $41,600-$51,999 (i.e., $800-$999 a week) |  |
| **9** | $52,000-$64,999 (i.e., $1,000-$1,249 a week) |  |
| **10** | $65,000-$77,999 (i.e., $1,250-$1,499 a week) |  |
| **11** | $78,000-$90,999 (i.e., $1,500-$1,749 a week) |  |
| **12** | $91,000-$103,999 (i.e., $1,750-$1,999 a week) |  |
| **13** | $104,000-$129,999 (i.e., $2,000-$2,499 a week) |  |
| **14** | $130,000-$155,999 (i.e., $2,500-$2,999 a week) |  |
| **15** | $156,000-$181,999 (i.e., $3,000-$3,499 a week) |  |
| **16** | $182,000-$207,999 (i.e., $3,500-$3,999 a week) |  |
| **17** | $208,000-$233,999 (i.e., $4,000-$4,499 a week) |  |
| **18** | $234,000-$259,999 (i.e., $4,500-$4,999 a week) |  |
| **19** | $260,000-$311,999 (i.e., $5,000-$5,999 a week) |  |
| **20** | $312,000-$415,999 (i.e., $6,000-$7,999 a week) |  |
| **21** | $416,000 or more (i.e., $8,000 or more a week) |  |
| **22** | Prefer not to answer |  |

### Income alternative [SINGLE]

#### Thinking about your current needs and financial responsibilities, would you say you are…?

##### Please select one answer

| **1** | Very poor |  |
| --- | --- | --- |
| **2** | Poor |  |
| **3** | Just getting along |  |
| **4** | Reasonably comfortable |  |
| **5** | Very comfortable |  |
| **6** | Prosperous |  |
| **7** | Prefer not to answer |  |

### 17 Education [SINGLE]

#### What is the highest level of education you have attained?

##### Please select one answer

| **1** | Year 11 or below |  |
| --- | --- | --- |
| **2** | Year 12 |  |
| **3** | Certificate III/IV |  |
| **4** | Bachelor’s Degree |  |
| **5** | Graduate Diploma or Graduate Certificate |  |
| **6** | Post graduate level (Masters or PhD) |  |
| **7** | Prefer not to answer |  |

### Ethnicity [SINGLE]

#### With which ethnic group do you identify?

##### Please select one answer

| **1** | Australian |  |
| --- | --- | --- |
| **2** | Indigenous Australian or Torres Strait Islander |  |
| **3** | New Zealander |  |
| **4** | Māori |  |
| **5** | Samoan |  |
| **6** | Tongan |  |
| **7** | Cook Islander |  |
| **8** | Niuean |  |
| **9** | Fijian |  |
| **10** | Other Pacific (please specify) |  |
| **11** | Pacific Islander |  |
| **12** | Asian |  |
| **13** | Indian |  |
| **14** | Middle Eastern |  |
| **15** | European |  |
| **16** | North American |  |
| **17** | South American |  |
| **18** | African |  |
| **19** | Other (please specify) | OTHER |
| **20** | Prefer not to answer |  |

# Feedback and thank you

## Feedback

### Feedback

#### Do you have any comments or suggestions for future surveys like this?

##### Please type in the box below

| OPEN TEXT BOX |
| --- |

### Reimbursement [SINGLE] – hidden for panel

#### In appreciation of your time and contribution, you can choose to either receive a $75 gift card, or have this amount donated to a support group on your behalf.

##### Please choose which option you would prefer

| **1** | E-Gift card | Use email address provided earlier: ^PIPE EMAIL^ |
| --- | --- | --- |
| **2** | Donation to CLL support group (i.e. Lymphoma Australia or Leukaemia Foundation) |  |

IF J2:1

### Reimbursement – hidden for panel

**You have selected to receive a $75 E-giftcard**

| **1** | Use previously provided email address: PIPE EMAIL ADDRESS |  |
| --- | --- | --- |
| **2** | Use another email address | Please collect email address |

### Recontact consent [SINGLE] – hidden for panel

**Do you give permission to be recontacted to participate in future research?**

##### Please select one answer

| **1** | Yes, I give permission for my email to be stored so I can be recontacted for future research |  |
| --- | --- | --- |
| **2** | No, I do not give permission for my email to be stored, so I can be recontacted for future research |  |

### Treatment Preference / Goals research – hidden for panel

**As part of the next stage of this research, there is another survey that looks to understand your treatment preferences for CLL and also what outcomes you are hoping to achieve with CLL treatment. This will also involve a 30-40 minute on-line survey and you will be paid for your time. Please indicate below if you are happy to take part in this component of research (if so, we will send you the on-line survey in mid July 2022).**

##### Please choose which option you would prefer

| **1** | Yes (this will be sent to the email address you provided earlier) | Use email address provided earlier: ^PIPE EMAIL^ |
| --- | --- | --- |
| **2** | No |  |

### Thank you

#### Thank you on behalf of Community and Patient Preference Research for taking part in the survey.

#### If you have any questions about the survey, please contact Rose Wilson at [rose.wilson@cappre.com.au](mailto:rose.wilson@cappre.com.au) or call 0467 952 347.

#### This research will help improve understanding of how patients experience the healthcare, including areas of satisfaction and dissatisfaction. The results will be used to guide future research into positive healthcare changes.

#### Remember to please see your doctor if you have any questions about your condition and its management.

#### Talking about your experience may raise aspects or emotions that could potentially cause you some distress. If this happens and you need support, or you feel that your CLL is not being adequately treated, you are advised to contact the healthcare professional treating your CLL or call one of the following hotlines:

- Leukaemia Foundation Australia 1800 620 420
- Lymphoma Care Nurse Support Line 1800 953 081

#### If you need urgent help, you can attend the emergency department at your local public hospital or call 000

#### Please click on "Next" button to close the survey. Please click "Next" to close the survey
