# Supplementary material for: Understanding the experience, treatment preferences and goals of people living with chronic lymphocytic leukemia (CLL) in Australia
Source: BMC Cancer. 2024 Jul 11;24:831. doi: 10.1186/s12885-024-12589-9 (PMC11241996; doi:10.1186/s12885-024-12589-9)
Supplement: Supplementary file 3 — Supplementary Material 3 [file 12885_2024_12589_MOESM3_ESM.docx]

## [Landing Page Heading]: Treatment Preferences and Goals of people living with a specific type of cancer

**Thank you for expressing interest in our research project! Your time and input is greatly appreciated.**

**The next few pages will include some questions that will help us determine your eligibility for the survey (which will take approximately 30-40 minute to complete). Sometimes we have people trying to complete the survey who are not suitable, which will be picked up when we audit our data. In order to prevent this from occurring, we may need to ask you some additional questions, so we accurately capture feedback from those with the condition. The payment of $70 will only be paid to suitable and genuine participants who complete the full survey. If you are eligible, you will be taken to an Information Sheet and Consent Form. If you choose to participate, you will then be taken directly to the main survey.**

# Section A: Diagnosis Screener

## About you

### Device [SINGLE]

#### What type of device are you using to complete the survey?

##### Please select one answer

| **1** | Desktop computer |  |
| --- | --- | --- |
| **2** | Laptop computer |  |
| **3** | Standard tablet size |  |
| **4** | Mini tablet | ERROR |
| **5** | Mobile phone | ERROR |

IF A1=4 or A1=5 RAISE ERROR: Thank you so much for taking the time to click though to the survey. Unfortunately mini tablets and mobile phones are too small to display the whole survey. If you can, please complete the survey on a desktop computer, laptop or standard-sized tablet.

### Email [OPEN TEXT] – hidden for panels

#### Thank you for expressing interest in our research project. Please enter your email address below to receive a personalised link to the survey (this email will be coming from CaPPRe). If at any point you need to take a break from the survey, you can stop and use the link sent to your email to continue from where you left off. *Please note we will not pass on your email or use it for any other purposes than for this study.*

##### If you do not receive an email, please check your “Junk” or “spam” folders.

##### Please enter a valid email address

|  | OPEN TEXT BOX | ERROR EMAIL INVALID |
| --- | --- | --- |

ERROR MESSAGE: “Please provide a valid email address.”

### Age [SINGLE]

#### Which group describes your age?

##### Please select one answer

| **1** | Less than 18 | SCREEN OUT |
| --- | --- | --- |
| **2** | 18-30 |  |
| **3** | 31-40 |  |
| **4** | 41-50 |  |
| **5** | 51-60 |  |
| **6** | 61-70 |  |
| **7** | 71-80 |  |
| **8** | 81 or older |  |
| **9** | Prefer not to answer |  |

### Residency [SINGLE]

#### Are you an Australian citizen or permanent resident?

##### Please select one answer

| **1** | Yes |  |
| --- | --- | --- |
| **2** | No | SCREEN OUT |

### Pharma [SINGLE]

#### Are you an employee of a pharmaceutical or medical devices company?

##### Please select one answer

| **1** | Yes | SCREEN OUT |
| --- | --- | --- |
| **2** | No |  |

### Diagnosis [MULTI; RANDOM ORDER]

#### Have you been diagnosed with any of the following by a medical professional?

##### Please select all that apply

| **1** | Chronic Lymphocytic Leukaemia | REQUIRED |
| --- | --- | --- |
| **2** | Secusitic Leukaemia | SCREEN OUT |
| **3** | Follicular Lymphoma | SCREEN OUT  IF A6:1 NOT SELECTED |
| **4** | Lymphoblastic Lymphoma |  |
| **5** | Melanoma |  |
| **6** | Multiple Myeloma |  |
| **7** | None of the above | SCREEN OUT |

### CLL Treatment Experience

#### This survey is specifically about your Chronic Lymphocytic Leukaemia (CLL). Have you ever received treatment (i.e. prescribed by your doctor) for CLL?

##### Please select one answer

| **1** | Yes, I am currently receiving treatment for my CLL | ASK A8a |
| --- | --- | --- |
| **2** | Yes, I have previously received treatment for CLL, but not at the moment | ASK A8b |
| **3** | No, I have never received treatment for CLL (i.e. watch and wait) | GO TO A9 |
| **4** | Don’t know / not sure | SCREEN OUT |

### A8a. Treatment Status [ASK IF A7: 1; SINGLE]

#### What ‘line of treatment’ are you currently on for CLL? Please refer to the definition provided below.

A line of treatment refers to how many different times you’ve had to start a new treatment for CLL. The first time you start treatment it is called first-line treatment. Second-line treatment is when you start treatment again after a time of remission or, if your first-line treatment did not work etc.

##### Please select one answer

| **1** | I am currently on 1^st^-line treatment |  |
| --- | --- | --- |
| **2** | I am currently on 2^nd^-line treatment |  |
| **3** | I am currently on 3^rd^ or 4^th^-line treatment |  |
| **4** | Other (please state) |  |
| **5** | Don’t know | SCREEN OUT |

### A8b:Treatment Status [ASK IF A7: 2; SINGLE]

#### How many ‘lines of treatment’ have you had for CLL? Please refer to the definition provided below.

A line of treatment refers to how many different times you’ve had to start a new treatment for CLL. The first time you start treatment it is called first-line treatment. Second-line treatment is when you start treatment again after a time of remission or, if your first-line treatment did not work etc.

##### Please select one answer

| **1** | I am not currently on treatment after having received 1st-line treatment |  |
| --- | --- | --- |
| **2** | I am not currently on treatment after having received 2^nd^-line treatment |  |
| **3** | I am not currently on treatment after having received 3^rd^ or 4^th^ -line treatment |  |
| **4** | Other (please state) |  |
| **5** | Don’t know | SCREEN OUT |

### A9: Mild to moderate side effects [SINGLE: RANDOMISE]

**Below is a list of potential side-effects you can get from treatment for CLL. Most of these are mild to moderate. Please tick which one would be the most unacceptable to you.**

##### Please select one answer

| **1** | Diarrhoea |  |
| --- | --- | --- |
| **2** | Fatigue |  |
| **3** | Muscle and bone pain |  |
| **4** | Bruising |  |
| **5** | Rash |  |
| **6** | Headaches |  |
| **7** | Infections |  |
| **8** | Increased blood pressure |  |
| **9** | Other (please specify) |  |

### A10: Out of pocket costs [A7:1 and 2: OPEN TEXT]

#### How much of your own money have you had to spend to have each line of treatment? (this includes all associated costs such as parking, transport, treatment). If you’re not sure, please give your best guess.

A line of treatment refers to how many different times you’ve had to start a new treatment for CLL. The first time you start treatment it is called first-line treatment. Second-line treatment is when you start treatment again after a time of remission or, if your first-line treatment did not work etc.

##### Please enter your response

| **1** | 1^st^-line | $ |
| --- | --- | --- |
| **2** | 2^nd^-line | $ |
| **3** | 3^rd^/4^th^ line | $ |
| **4** | Don’t know | SCREEN OUT |

### A11: Check 1: Diagnostic tests [OPEN TEXT]

#### If you can recall, what type of test(s) were used to confirm your diagnosis of CLL?

##### Please enter your response

|  | OPEN TEXT BOX |  |
| --- | --- | --- |

PROGRAMMER INSTUCTIONS: POTENTIAL TESTS INCLUDE: COMPLETE BLOOD COUNT [CBC – USUALLY SHOWING AN ABNORMALLY HIGH NUMBER OF UNUSUAL WHITE BLOOD CELLS (LYMPHOCYTES)], TESTS ON BLOOD, BONE MARROW OR FLUID SAMPLE (CALLED FLOW CYTOMETRY), CT / PET SCANS].

### A12: Check 2: Key symptoms [OPEN TEXT]

#### Please describe the most troublesome symptom(s) you have experienced due to CLL?

##### Please enter your response

|  | OPEN TEXT BOX |  |
| --- | --- | --- |

### A13: Check 3a: Current treatment [IF A7:1; OPEN TEXT]

#### What treatment are you currently having for CLL?

##### Please enter your response

|  | OPEN TEXT BOX |  |
| --- | --- | --- |

### A14: Check 3b: Past treatment [IF A7:2; 8a:2 and 3: OPEN TEXT]

#### What treatments have you had in the past for CLL?

##### Please enter your response

|  | OPEN TEXT BOX |  |
| --- | --- | --- |

### A15: Check 4: Key hospital [OPEN TEXT]

**Which hospital or clinic do you go to for the management of your CLL? If more than one, which is the main one?**

##### Please enter your response

|  | OPEN TEXT BOX |  |
| --- | --- | --- |

### A16: Check 5: Treating doctor [OPEN TEXT]

**What is your treating doctor’s name? (**i.e. **your main haematologist or oncologist looking after your CLL)**. Please be assured we won’t contact your doctor.

##### Please enter your response

|  | OPEN TEXT BOX |  |
| --- | --- | --- |

Screen out text: “Unfortunately, you do not qualify for this study. Thank you for your time and interest in the study.”

Screen in text:

We are pleased to inform you that you qualify to participate in this research project. Thank you for your time and interest!

You will now be shown to the information sheet, consent form and survey. You are free to withdraw participation at any time by exiting the survey window.

Press “Next” to continue

# Section B: Participation Information Form & Consent

**Protocol Number:** PCSONC00442, Version 3, 7th June, 2022: Page 1 of 5

| **Study Title:** | | Chronic Lymphocytic Leukaemia Treatment Preference Research |
| --- | --- | --- |
| **Principal Investigator (Study Doctor):** | | Dr Simon Fifer |
| **Telephone:** | | +61 403 862 091 |
| **Local Legal Entity Name and Address:** | | Community and Patient Preference Research (CaPPRe): Level 20, 25 Bligh Street, Sydney Australia |
| **Study Sponsor** |  | CaPPRe |
| **Funder of Study** |  | Janssen ANZ |

## Introduction

This Information Sheet tells you about the research project. It explains the purpose of the research and how the information you provide will be used and shared. Please read this information carefully. Participation in this research is voluntary. If you decide you would like to take part, you will be asked to sign the Consent Form. By signing it you are telling us that you;

- Understand what you have read
- Agree to take part in the research project
- Consent to the use of your personal information as described
- Understand that the funding for this research has been provided by a pharmaceutical company and that there is no conflict of interest between the funder and all other relevant parties involved.

If you would like a copy of this participant information and consent form to keep, click on print or you can take screenshots of these pages and save to your computer.

**Protocol Number:** PCSONC00442, Version 3, 7^th^ June, 2022: Page 2 of 5

**PURPOSE OF THE STUDY**

Community and Patient Preference Research (CaPPRe) is working collaboratively with the pharmaceutical company, Janssen ANZ, who is funding this research on CLL. This study will include about 30 people who have been diagnosed with CLL. We hope to gain a better understanding of the experiences of people with CLL, with a focus on treatment goals. We also want to better understand what is important to you regarding CLL treatment, and the long-term outcomes.

CaPPRe is a company that specialises in conducting research to learn about people’s preferences. We want to understand what people believe are valuable aspects of different treatments available for their conditions. This type of research is called ‘Treatment Preference Research’. We hope this will help in the assessment of new treatment/s as we learn more about patient preferences regarding potential side-effects, and ways to have treatment.

We also specialise in understanding the overall patient journey which includes what type of outcomes people are hoping to achieve with their CLL treatment.

**WHAT’S INVOLVED?**

We would like to invite you to participate in an online survey where you will be asked some questions about treatment you have taken for CLL and the long-term goals that you most value. You will be shown several scenarios for different proposed treatment options and asked to choose which treatment option you prefer. You will also be shown several scenarios that cover long-term treatment and life goals that may be important to you. It is ok to have your primary carer help you complete the survey if needed (i.e. to assist with the on-line component and to explain what you need to do – but they cannot answer the questions for you).

**HOW LONG WILL IT TAKE?**

The survey should take about 30-40 minutes to complete.

**WHAT DEVICE SHOULD I USE TO COMPLETE THE SURVEY?**

You should use a device with a screen size of at least 10 inches in size (e.g., desktop computer, laptop computer or standard size tablet).

**QUESTIONS OF A SENSITIVE NATURE**

If there is anything in the survey that you feel uncomfortable answering, you can withdraw your participation at any time. We have provided contact numbers for patient support below. We also encourage you to speak to your treating healthcare professional/s if required.

- Leukaemia Foundation Australia 1800 620 420 (8.30am-5.00pm, AEST; Monday-Friday)
- Lymphoma Care Nurse Support Line 1800 953 081

**Protocol Number:** PCSONC00442, Version 3, 7^th^ June, 2022: Page 3 of 5

**POTENTIAL BENEFITS AND RISKS**

Taking part in this research will help improve understanding about perspectives on the benefits and challenges of the treatment journey in CLL. The results may be used in future discussions with government about new treatments as

well as informing further research into ‘Treatment Preferences’ which may be published so that others can learn from them. Since this research deals with the collection and use of data / information only, it is not anticipated that the research will directly benefit you.

While care has been taken in designing this survey, a risk for you is the potential to experience some emotional burden as a result of your participation. As mentioned above, if there is anything you feel uncomfortable answering, you can withdraw your participation at any time. We encourage you to seek support from patient support groups or your doctor.

**SUMMARY OF FINDINGS**

A summary of study findings will be available to participants following completion of the entire project. If you would like to receive a summary of the findings, we will collect your email address so we can send you the results. Your privacy is important to us, so we will not use your email for any other purpose. We will also delete your email from our database once we have sent you the information.

**PAYMENT** [HIDE FOR PANELS]

By way of appreciation for your time and participation you will receive $70 upon completion of the full survey. This will be paid via an e-gift card. Alternatively, you could choose to donate the amount to a CLL patient support group. Prior to payment, all responses will be individually assessed to determine their sincerity. To ensure responses are genuine, you may be contacted to clarify some of your responses and provide further information – payment will not be made for suspected fraudulent participation.

**VOLUNTARY PARTICIPATION / RIGHT TO REFUSE OR WITHDRAW**

There is no obligation for you to be involved in this research and if you decide to participate, you may withdraw from the research at any time. You will not be disadvantaged if you do not take part in the survey, or if you decide not to take part at any point. Please note that once you have agreed to take part in the survey, if you choose to discontinue at any point, no new information will be collected. However, the information you have provided up to the point of withdrawing will be used in data analysis (unless you tell us that you do not wish it to be included in data analysis by contacting [jenni.godsell@cappre.com.au](mailto:jenni.godsell@cappre.com.au) or calling 0468 435 926.

**CONFIDENTIALITY**

CaPPRe respects your privacy. We ask you for some information about yourself and your treatment history. Your personal details will not be forwarded to any other parties, nor will you be contacted by CaPPRe for anything other than this research project unless you choose to be. All responses are treated with the strictest confidentiality. CaPPRe alone will know which people have participated and their responses. No individual data will be available to anyone other than CaPPRe. There is no collection, transmission, analysis, storage and/or security of data outside of Australia at any time-point. It is anticipated that the results of this research project will be published and/or presented in a variety of forums. In any publication and/or presentation, information will be provided in a combined form such that no individual person can be identified. Our client will be provided with a report of the findings containing only combined de-identified data. The research data will be kept securely for a period of 5 years.

**Protocol Number:** PCSONC00442, Version 3, 7^th^ June, 2022: Page 4 of 5

**CONTACT INFORMATION**

If you have any questions or concerns you can contact [jenni.godsell@cappre.com.au](mailto:jenni.godsell@cappre.com.au) or call 0468 435 926.

**CODE OF RESEARCH CONDUCT**

The Bellberry Human Research Ethics Committee has reviewed and approved this study in accordance with the National Statement on Ethical Conduct in Human Research (2007) incorporating all updates. This Statement has been developed to protect the interests of people who agree to participate in human research studies. Should you wish to discuss the study or view a copy of the Complaint procedure with someone not directly involved, particularly in relation to matters concerning policies, information or complaints about the conduct of the study or your rights as a participant, you may contact the Operations Manager, Bellberry Human Research Ethics Committee on 08 8361 3222.

**REPORTABLE ADVERSE EVENTS INCLUDING DRUG SIDE EFFECTS**

If during the survey, you report a side effect (Adverse Event), special situation or product quality issue that you have experienced while taking a medicine from the pharmaceutical company funding this research, there is an obligation by the pharmaceutical company to report this as part of the ongoing post-market monitoring of their product. This is a mandatory requirement by the pharmaceutical company regarding its own products and therefore, any information you provide during the survey relating to their specific side effects will need to be sent to the pharmaceutical company for their records.

In this instance, you will be asked whether or not you are willing to waive the confidentiality given to you under the Market Research Codes of conduct specifically in relation to that adverse event/product quality complaint. Everything else you say during the course of the survey will continue to remain confidential, and you will still have the option to remain anonymous if you so wish. Please note that we are only reporting side effects of products from the pharmaceutical company funding this research. If you have concerns regarding side effects with any product you have received for CLL, we recommend that you seek advice from your GP or specialist.

### B1: AE [SINGLE]

#### Please tell us what information you permit us to share with the sponsoring company’s Drug Safety Department if you mention an Adverse Event or Product Quality Complaint associated with their products.

##### Please select one answer

| **1** | **My de-identified information** (e.g. gender) **with my contact details** (you may be recontacted by the sponsoring companies Drug Safety Department for more information) | Request email address **Hidden for panels** |
| --- | --- | --- |
| **2** | **My de-identified information** (e.g. gender) but **without my contact details** (i.e. anonymously, you will not be recontacted by the sponsoring drug company for more information) |  |
| **3** | **No information,** I understand **I will not be able to participate in this research** | SCREEN OUT |

### B2: Email address [IF B1:1 OPEN TEXT]

#### Please confirm your email address.

##### Please note that your email will only be used for the purpose specified

|  | OPEN TEXT BOX |  |
| --- | --- | --- |

##### Please click "Next" to continue

**Protocol Number:** PCSONC00442, Version 3, 7^th^ June, 2022: Page 5 of 5

## Consent

I hereby consent to my involvement in this research project.

I acknowledge that the nature, purpose and risks of the research project and alternatives to participation have been explained. Specifically, the details of the research and the anticipated length of time it will take to complete the survey have been communicated.

- I am 18 years of age or over
- I freely agree to participate in this research project per the conditions in the Participant Information Sheet.
- I understand that my involvement in this research may not be of any direct benefit to me.
- I have been given the opportunity to discuss the project with a member of my family or another person.
- I have been told that neither my personal information nor information regarding my medical history will be passed on to unauthorised third parties and the findings from this research will not include any identifying information in publication or other forms of dissemination.
- I understand that I am free to withdraw from the research at any stage. If I decide to withdraw from the research, I agree that the information collected about me up to the point when I withdraw may continue to be processed.
- I do not have a prior or current relationship with CaPPRe.
- I declare that all my questions have been answered to my satisfaction.
- I understand that the results may be used for commercial purposes.
- I understand that the funding for the research is provided by a pharmaceutical company and that there is no conflict of interest between the funder and all other parties involved.

### ****B3: Consent**** [SINGLE]

#### I have read, or have had read to me, and I understand the Participant Information Sheet, dated [INSERT DATE].

##### Please select one answer

| **1** | I consent voluntarily to take part in this research |  |
| --- | --- | --- |
| **2** | I do not consent to be a part of this research | SCREEN OUT |

##### Please remember to print / save screens related to the participant information, your consent to AE reporting and your consent to participate in this research if you would like to keep a record of this information.

Please keep in mind that if at any point you need to take a break from the survey, you can stop and use the link sent to your email to continue from where you left off. It is acceptable and appropriate for a primary carer to be involved in assisting you complete the survey if required (i.e. to assist with the on-line component and explanations of survey requirements). Should you have any questions, please contact [jenni.godsell@cappre.com.au](mailto:jenni.godsell@cappre.com.au) or call 0468 435 926.

### ****B4a: Attention [SINGLE]****

#### Paying attention and reading the instructions carefully is really important for this survey. If you are paying attention, please choose the answer option “Moderately disagree” below.

##### Please select one answer

| **1** | Strongly agree |  |
| --- | --- | --- |
| **2** | Moderately agree |  |
| **3** | Somewhat agree |  |
| **4** | Neither agree nor disagree |  |
| **5** | Somewhat disagree |  |
| **6** | Moderately disagree | IF NOT SELECTED: ASK QUESTION B4b |
| **7** | Strongly disagree |  |
| **8** | Don’t know |  |

### ****B4b: Attention [IF NOT B4a:6; SINGLE]****

#### Paying attention and reading the instructions carefully is really important for this survey. If you are paying attention, please choose the answer option “Somewhat agree” below.

##### Please select one answer

| **1** | Strongly agree |  |
| --- | --- | --- |
| **2** | Moderately agree |  |
| **3** | Somewhat agree | IF NOT SELECTED: SCREEN OUT |
| **4** | Neither agree nor disagree |  |
| **5** | Somewhat disagree |  |
| **6** | Moderately disagree |  |
| **7** | Strongly disagree |  |
| **8** | Don’t know |  |

Screen out text if failed attention test: “Unfortunately, your response or a combination of your responses was flagged by our data auditing team, and you now do not qualify for this study. Thank you for your time and interest in the study.

# Section C: Treatment Preferences

**In this section of the survey, we would like to understand your preferences when it comes to CLL treatment**

Please imagine your doctor has recommended your CLL treatment status be reviewed and that there is a choice between the treatments available. Please indicate which treatment option would be your preference. The answers to these questions will tell us which factors are most important to you when choosing between treatments. There are no right or wrong answers. We are just interested in your preferences.

You will be shown 10 screens which will ask you to choose your preferred treatment from the options given. Please note that these may not necessarily reflect actual treatments for CLL (i.e. they are proposed examples to illustrate some of the factors that are associated with treatments). Some of the scenarios may seem similar but there are small differences between them.

On each screen, you will be asked to compare two treatments (A and B). When indicating your preferred treatment, you can choose one of the treatments presented or ‘neither of these’. The ‘neither of these’ option does not mean no treatment, but rather that you would not choose either of the two treatments shown in the scenarios.

When looking at each scenario, please consider the attributes together as this will enable you to form the complete picture of that specific treatment option.

The treatments will be described by the factors listed below:

1. **Type of treatment:** This can be a needle into the vein [Intravenous (IV) infusion], tablets or a combination of needle (IV) and tablets.
   1. Treatment involving a needle into the vein (IV) is received in hospital usually every month for 6 months
   2. Tablets are usually taken daily at home for a fixed time or ongoing
   3. Combination treatment involves a needle into the vein (i.e. IV in hospital every month for 6 months) plus daily tablets (for a fixed time or ongoing)
2. **How long you stay on treatment:** this can be for a fixed time or ongoing

- Fixed time periods can include: 6 months (i.e. treatment with needle in the vein every month), daily tablets for 15 months or daily tablets for 24 months
- Ongoing treatment: daily tablets [with or without treatment with needle in the vein (IV every month for 6 months)] until CLL progresses or unacceptable side effects are experienced

1. **Average time until your CLL progresses:** This refers to the length of time during and after a CLL treatment that you live with CLL but the condition is stable / not getting worse
2. **Mild to moderate side effects:** The chance of experience mild to moderate side effects with your CLL treatment. As a reference point, please consider the chance of you experiencing (PROGRAMMER: PIPE: A9) as a mild to moderate side effect.
3. **Severe side effects:** The chance of experiencing severe side effects with your CLL treatment (i.e. side effects that are unacceptable and that significantly interfere with daily life. These side effects are likely to require additional treatment and/or hospitalisation or may lead to having to stop that treatment). Examples of severe side effects could include severe infection [e.g. pneumonia], haemorrhage (i.e. bleeding), heart issues (e.g. atrial fibrillation or heart failure).
4. **Long term risk:** The chance of experiencing a long-term risk with your CLL treatment. Examples of long-term risks could include organ damage (e.g. kidneys, liver, heart, bone marrow), secondary cancers (e.g. skin cancer), increased blood pressure.
5. **Average yearly out of pocket costs:** This refers to average costs over one year that you spend due to CLL (e.g. travel, doctor costs, hospital costs). This does not include treatment costs as these are covered by the government (Medicare).

* The chance of something occurring (side effects/long term effects) will be expressed as a percentage. For example:


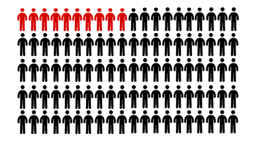


**10% chance**: this means that 10% of people (or 10 out of every 100 people) taking the medicine will experience the side effects/long term effects.

**DCE UNDERSTANDING CHECK 1:**

Due to the complexity of this survey, we would like to check that our explanations of the different treatment features have been interpreted as we intended. Therefore, please review the following two options and select the one you think would provide you with the most suitable result.

If you consider the following two treatment options, which one would provide you with the most suitable result?

|  | **Option A** | **Option B** |
| --- | --- | --- |
| **Chance of severe side effects** | 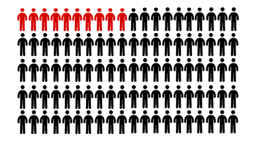  **10% chance** | 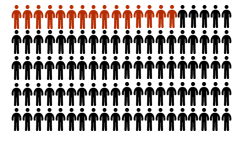  **15% chance** |

IF Q1 OPTION B CHOSEN: We noticed you chose Option B, however the treatment that would provide you with the most suitable result (the lowest chance of severe side effects) would be Option A since a 10% chance is lower than a 15% chance. Please keep this in mind when you consider the upcoming treatment options.

IF Q1 OPTION A CHOSEN: This is correct. Please keep this in mind when you consider the upcoming treatment options.

**DCE UNDERSTANDING CHECK 2:**

Please review the following two options and select the one you think would provide you with the most suitable result.

|  | **Option A** | **Option B** |
| --- | --- | --- |
| **Long term risk** | **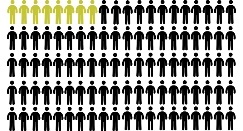**  **8% chance** | **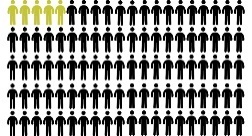**  **5% chance** |

If you consider the following two treatment options, which one would provide you with the most suitable result?

IF Q2 OPTION A CHOSEN: We noticed you chose Option A, however the treatment that would provide you with the most suitable result (the lowest long term risk) would be Option B since a 5% chance is lower than an 8% chance. Please keep this in mind when you consider the upcoming treatment options.

IF Q2 OPTION B CHOSEN: This is correct. Please keep this in mind when you consider the upcoming treatment options.

PROGRAMMING INSTRUCTIONS: Example of a choice task:

Scenario 7 of 12: Please review the following treatment options and choose the treatment option you would prefer.


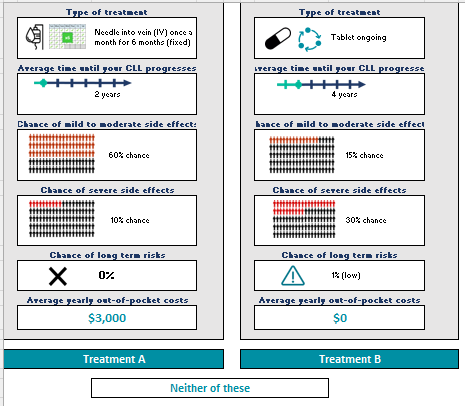


**DCE Attributes and Levels**


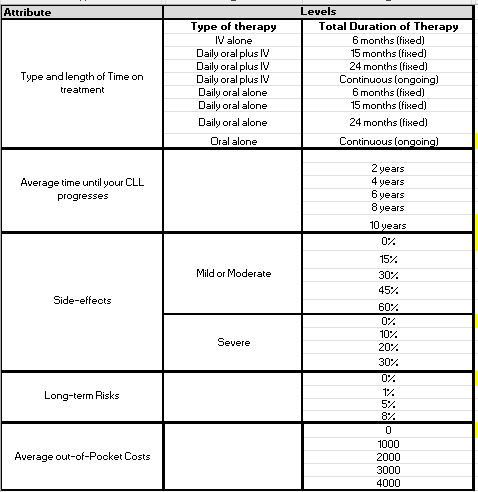


# Section D: DCE feedback

## In the next section of the survey we would like to get your feedback about the scenarios you have just completed

### D1: Understanding [SINGLE]

##### Please rate your understanding of the scenarios you have just completed on a scale from 1 ("Did not understand the scenarios at all") to 10 ("Completely understood the scenarios"). Please select one answer

| 1-10 SCALE |
| --- |

### D2: Reflect real choices

#### How well do you think your choices for each scenario reflect what you would do in real life (1= “Not at all” and 10 = “Completely”)?

#### Please select one answer

| 1-10 SCALE |
| --- |

### D3: Most/least [OPEN]

##### For you, what were the most and least important treatment factors in these scenarios? Please explain your reasons for this. Please select an option for each row and provide reasons for your answers

|  |  |  | **Reason why** |
| --- | --- | --- | --- |
| **1** | **Most important:** | **[Drop down list of attributes]** |  |
| **2** | **Least important:** | **[Drop down list of attributes]** |  |

D4: Information [3D GRID, MULTI + MULTI; INTERNAL USE ONLY]

#### For each of the features used to describe the options during the scenarios, please indicate:

#### Whether the information provided for this feature easy to understand

#### Whether you would have liked any additional information for this feature

##### Please select an answer for each feature

|  |  | Information was easy to understand | Required additional information |
| --- | --- | --- | --- |
| **1** | Attributes |  |  |

### D5: All none explanation [OPEN TEXT] – ASK IF ALL NONE IN DCE

#### You chose neither of these for all scenarios. Can you please explain why you never chose one of the proposed treatment options?

##### Please enter your response

| OPEN TEXT BOX |
| --- |

# Section E: Further Treatment Preference Questions

**In this section of the survey, we would like to ask a couple more questions about your treatment preferences for CLL**.

### E1: Frequency and number of tablets preference [SINGLE]

#### Thinking about the number of tablets and how often you would need to take a tablet as treatment for CLL, please let us know your preference from the two options below:

##### Please select one answer

| **1** | I would prefer to take 3 tablets once a day |  |
| --- | --- | --- |
| **2** | I would prefer to take 1 tablet twice a day |  |
| **3** | Don’t know |  |

### E2: Reasons for preference [OPEN TEXT]

#### Please let us know the reasons for your preference.

##### Please enter response

| OPEN TEXT |
| --- |

# Section F: Best Worst Scale (BWS)

**In this section of the survey, we would like to understand what is most important to you personally when it comes to long-term goals of having treatment for CLL**.

On the next 11 screens, you will be shown a number of long-term goals with treatment for CLL. Some of the screens may seem similar but there are small differences between them. Please take a moment to take a look at the items in each set on each screen.

Thinking about the next 2 to 3 years, which goal is the **MOST IMPORTANT** and which goal is the **LEAST IMPORTANT** to you personally in each set.

If you are struggling to choose a statement from one of the columns, please try to select the statement that is most appropriate. For example, if all 6 statements shown are important to you and you are struggling to choose one for the 'least important' column, please select the one that is the least important out of the 6 shown. You will be given the chance to justify your answers at the end of the task. You will be given the chance to justify your answers at the end of the task.

BWS ITEM LIST: Insert according to grid

|  | **GOALS STATEMENTS** | **Description (Hover-over text)** |
| --- | --- | --- |
| **1** | **Having an income and / or work** | Not being restricted in my ability to work or earn an income due to difficulties that arise from CLL |
| **2** | **Being physically healthy** | Being physically fit and healthy (i.e. ability to exercise and participate in physical activities) |
| **3** | **Living a long life** | Having a long life expectancy despite having CLL |
| **4** | **Spending time with my family / friends** | Interacting / having personal relationships with my family and friends (e.g. socialising, doing things together) |
| **5** | **Pursuing my interests in life** | Engaging in my hobbies (e.g. travelling, going out to restaurants) |
| **6** | **Being independent** | Having autonomy (i.e. not having to rely on others) |
| **7** | **Feeling well emotionally** | Feeling happy and positive; reduced concern / worry that arises from CLL (e.g. fear for the future) |
| **8** | **Avoiding hospitalisation** | Staying out of hospital [arising from CLL complications (e.g. infections)] |
| **9** | **Having financial wellbeing** | Not feeling pressured financially due to personal costs associated with CLL (e.g. doctor fees, travel to and from hospital / clinic, parking at hospital / clinic) |
| **10** | **Reducing my hospital / clinic visits** | Reducing the need for me to travel to the hospital / clinic (e.g. to have treatment or to see my specialist) |
| **11** | **Being able to stop ongoing treatment** | Being able to stop having CLL treatment on an on-going / continuous basis |

Please consider your personal thoughts with each of these potential CLL treatment goals. There are no right or wrong answers. We are purely interested in which goals are important to you.

PROGRAMMER: INCLUDE EXAMPLE OF VISUAL RESPRESENTATION ONCE SCRIPTED: E.G.

**
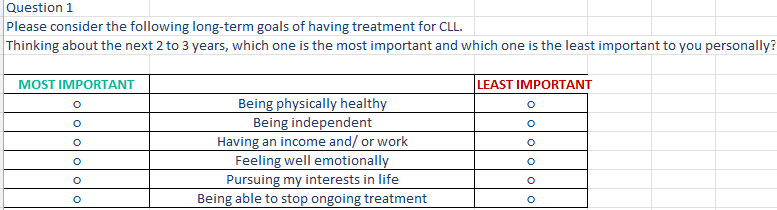
**

# Section G: BWS Task Feedback

## In the next section we’d like to get your feedback about the goals statements you have just considered

### G1: Most Importance probed [OPEN TEXT]

##### Based on the choice tasks in the last section of the survey, the 3 goals with CLL treatment that were most important to you included:

SHOW 3 MOST IMPORTANT ITEMS

##### Please explain your reasons why these goals with CLL treatment are most important to you.

##### Please enter your response for each

| **1** | PIPE: Importance statement rank 1 | OPEN TEXT |
| --- | --- | --- |
| **2** | PIPE: Importance statement rank 2 | OPEN TEXT |
| **3** | PIPE: Importance statement rank 3 | OPEN TEXT |

### G2: Understanding [SINGLE]

##### Please rate your understanding of the scenarios you have just completed on a scale from 1 ("Did not understand the scenarios at all") to 10 ("Completely understood the scenarios"). Please select one answer

| - 1. SCALE |
| --- |

### G3: Ease [SINGLE]

##### How easy did you find answering these scenarios on a scale from 1 ("Very Difficult") to 10 ("Very Easy")? Please select one answer

| - 1. SCALE |
| --- |

### G4: Feedback on goal statements [GRID]

#### Please select whether you understood the definitions for each treatment goal, or if they were not clear.

##### Please select an answer for each.

|  |  | **I understood this definition** | **This definition was not clear to me** |
| --- | --- | --- | --- |
|  | INSERT ACCORDING TO BWS DESIGN | Yes/no | Yes/no |

### G5: Other goals [OPEN TEXT]

#### Are there any other goals you have with CLL treatment that were not covered in the statements provided?

##### Please enter your response

| OPEN TEXT BOX |
| --- |

# Section H: Disease & Treatment Background

## Questions about your Chronic Lymphocytic Leukaemia (CLL)

### H1: Time since diagnosis [SINGLE]

#### How long ago were you diagnosed with CLL?

##### Numeric Drop Down

| **1** | Months | LIST 1-12 |
| --- | --- | --- |
| **2** | Years | LIST 90 |
| **3** | Don’t know |  |

### H2: Genetic Mutation [SINGLE]

#### If you are aware, do you have a genetic marker associated with your CLL diagnosis?

##### Please select one answer

| **1** | Yes |  |
| --- | --- | --- |
| **2** | No | GO TO H4 |
| **3** | Don’t know | GO TO H4 |

### H3: Genetic Mutation [IF H2: 1; SINGLE]

#### If you are aware, which genetic marker is associated with your CLL?

##### Please select all that apply

| **1** | 17p deletion |  |
| --- | --- | --- |
| **2** | 11p deletion |  |
| **3** | TP53 mutation |  |
| **4** | Unmutated IGHV |  |
| **5** | Other (please specify) |  |
| **6** | Don’t know |  |

### H4: Current Treatment Approach: Combination / Monotherapy [IF A7: 1 SINGLE]

#### We are interested to know the current treatment you are having for CLL. Please indicate if your treatment is a combination of products (i.e. more than one treatment at the same time) or if it is a single product (only one treatment).

##### Please select one answer

| **1** | I am having a combination of treatments for my CLL | GO TO H5 |
| --- | --- | --- |
| **2** | I am having a single treatment for my CLL | GO TO H6 |

### H5: Current Combination Treatment [IF H4: 1 SINGLE]

#### Please refer to the table below and to the best of your knowledge, select the combination of treatments you are currently taking for your CLL.

#### If you are on a combination of treatments that is not listed, please select ‘other’ and specify the combination you are on.

##### Please select one answer

| Combination Treatment for CLL | Current Treatment |
| --- | --- |
| 1. Bendamustine + Rituximab |  |
| 1. Bendamustine + Obinutuzumab |  |
| 1. Chlorambucil + Obinutuzumab |  |
| 1. Fludarabine + Cyclophosphamide + Rituximab (FCR) |  |
| 1. Idelalisib + Bendamustine |  |
| 1. Idelalisib + Rituximab |  |
| 1. Venetoclax + Obinutuzumab |  |
| 1. Venetoclax + Rituximab |  |
| 1. Other (please specify) |  |

### H6: Single Treatment [IF H4:2 SINGLE]

#### Please refer to the table below and to the best of your knowledge, select the treatment you are currently taking for CLL. If you are not on a treatment that is listed below, please select ‘other’ and specify the treatment you are on.

##### Please select one answer

| Single Treatment for CLL | Current Treatment |
| --- | --- |
| 1. Acalabrutinib |  |
| 1. Ibrutinib |  |
| 1. Venetoclax |  |
| 1. Other (please specify) |  |

### H7: Past Treatment Approaches [IF A7: 2 AND A8a: 2 & 3: MULTIPLE]

#### Please now refer to the table below and to the best of your knowledge, select all the treatments you have taken in the past for your CLL (we have included combination treatments and single treatments as options).

#### Please leave treatment options that you have not received blank.

##### If there are treatments that you have taken in the past that are not on the list, please indicate these treatments in the ‘other’ option

##### Please select all relevant options

| **Treatment Taken in the past for** **CLL** | | |
| --- | --- | --- |
| **Combination Treatment** | 1. Bendamustine + Rituximab |  |
|  | 1. Bendamustine + Obinutuzumab |  |
|  | 1. Chlorambucil + Obinutuzumab |  |
|  | 1. Fludarabine + Cyclophosphamide + Rituximab (FCR) |  |
|  | 1. Idelalisib + Bendamustine |  |
|  | 1. Idelalisib + Rituximab |  |
|  | 1. Venetoclax + Obinutuzumab |  |
|  | 1. Venetoclax + Rituximab |  |
| **Single Treatment** | 1. Acalabrutinib |  |
|  | 1. Ibrutinib |  |
|  | 1. Venetoclax |  |
|  | 1. Other (please specify combinations and /or single treatments) |  |

### H8: Co-morbidities [SINGLE]

#### Have you been diagnosed with any conditions (other than CLL) that you are taking treatment for?

##### Please select one answer

| **1** | Yes |  |
| --- | --- | --- |
| **2** | No | GO TO SECTION I |
| **3** | Don’t know | GO TO SECTION I |

### H9: Co-morbidities and Treatment [IF H8:1; MULTI, OPEN TEXT]

#### Please let us know which other conditions you have been diagnosed with and any treatment you are currently taking for these other conditions?

##### Please enter your response(s)

| **Condition(s)** | | **Treatment taken for condition(s)** |
| --- | --- | --- |
| **1** |  |  |
| **2** |  |  |
| **3** |  |  |

### H10: Use of Proton Pump Inhibitor (PPI)

#### Are you currently using treatment referred to as ‘Proton Pump Inhibitors’ as treatment for your other condition(s)? Please refer to information about Proton Pump Inhibitors below:

#### Proton pump inhibitors (PPIs) are medicines (tablets) that work by reducing the amount of stomach acid made by glands in the lining of your stomach. Proton Pump Inhibitors can be used to treat conditions including gastric (stomach) ulcers, duodenal (intestinal) ulcers, reflux esophagitis, and gastroesophageal reflux disease (GERD). Some examples of Proton Pump Inhibitors include: Omeprazole (Losec), Lansoprazole (Zopral), Esomeprazole (Nexium), Rabeprazole (Pariet), Pantoprazole (Somac).

##### Please select one answer

| **1** | Yes |  |
| --- | --- | --- |
| **2** | No | GO TO SECTION I |
| **3** | Don’t know | GO TO SECTION I |

### H11: Comfort Level in Changing / Stopping Use of Proton Pump Inhibitor (PPI) [IF H11:1; SCALE]

#### If your doctor recommended a CLL treatment that required you to either change your current Proton Pump Inhibitor treatment or stop using your Proton Pump Inhibitor treatment, how comfortable would you be to do this on a scale from 1 (“Not at all comfortable”) to 10 (“Completely comfortable”)

##### Please select one answer

| - 1. SCALE |
| --- |

### H12: Reasons for Comfort Level [OPEN TEXT]

##### Please describe the reason for this (i.e. your comfort level in changing or stopping your current Proton Pump Inhibitor treatment as a result of your CLL treatment)? Please enter your response

| OPEN TEXT BOX |
| --- |

# Section I: Demographics

## Background questions about you

### I1: Gender [SINGLE]

#### Which of the following best described your gender identity?

##### Please select one answer

| **1** | Male |  |
| --- | --- | --- |
| **2** | Female |  |
| **3** | Non-binary/gender fluid |  |
| **4** | Prefer to self-describe (please specify) | OTHER |
| **5** | Prefer not to answer |  |

### I2: State [SINGLE]

#### Which state/territory do you currently live in?

##### Please select one answer

| **1** | ACT |  |
| --- | --- | --- |
| **2** | NSW |  |
| **3** | VIC |  |
| **4** | QLD |  |
| **5** | SA |  |
| **6** | WA |  |
| **7** | TAS |  |
| **8** | NT |  |

### I3: Area [SINGLE]

#### How would you best describe the area you live in?

##### Please select one answer

| **1** | Metro/city |  |
| --- | --- | --- |
| **2** | Regional / Rural |  |

### I4: Household [SINGLE]

#### Which of the following best describes your household?

##### Please select one answer

| **1** | Couple with no children |  |
| --- | --- | --- |
| **2** | Couple family with children |  |
| **3** | One parent family |  |
| **4** | Single person household |  |
| **5** | Group household (i.e., shared) |  |
| **6** | Other (please specify) | OTHER |
| **7** | Prefer not to answer |  |

### I5: Occupation [SINGLE]

#### What is your occupation status?

##### Please select one answer

| **1** | Working (full-time) |  |
| --- | --- | --- |
| **2** | Working (part-time) |  |
| **3** | Working (casual) |  |
| **4** | Student |  |
| **5** | Not working |  |
| **6** | Home duties and/or caring responsibilities |  |
| **7** | Retired |  |
| **8** | Other (please specify) | OTHER |
| **9** | Prefer not to answer |  |

### I6: Occupation: [SINGLE; CAPTURE I5:2, 3,5 and 7)

#### Did your CLL cause you to reduce your work hours?

##### Please select one answer

| **1** | Yes |  |
| --- | --- | --- |
| **2** | No |  |

### I7: Education [SINGLE]

#### What is the highest level of education you have attained?

##### Please select one answer

| **1** | Year 11 or below |  |
| --- | --- | --- |
| **2** | Year 12 |  |
| **3** | Certificate III/IV |  |
| **4** | Bachelor’s Degree |  |
| **5** | Graduate Diploma or Graduate Certificate |  |
| **6** | Post graduate level (Masters or PhD) |  |
| **7** | Prefer not to answer |  |

### I8: Income [SINGLE]

#### Which of the following categories describes your annual total household gross income (before tax)?

##### Please select one answer

| **1** | Nil income |  |
| --- | --- | --- |
| **2** | $1-$7,799 (i.e., $1-$149 a week) |  |
| **3** | $7,800-$15,599 (i.e., $150-$299 a week) |  |
| **4** | $15,600-$20,799 (i.e., $300-$399 a week) |  |
| **5** | $20,800-$25,999 (i.e., $400-$499 a week) |  |
| **6** | $26,000-$33,799 (i.e., $500-$649 a week) |  |
| **7** | $33,800-$41,599 (i.e., $650-$799 a week) |  |
| **8** | $41,600-$51,999 (i.e., $800-$999 a week) |  |
| **9** | $52,000-$64,999 (i.e., $1,000-$1,249 a week) |  |
| **10** | $65,000-$77,999 (i.e., $1,250-$1,499 a week) |  |
| **11** | $78,000-$90,999 (i.e., $1,500-$1,749 a week) |  |
| **12** | $91,000-$103,999 (i.e., $1,750-$1,999 a week) |  |
| **13** | $104,000-$129,999 (i.e., $2,000-$2,499 a week) |  |
| **14** | $130,000-$155,999 (i.e., $2,500-$2,999 a week) |  |
| **15** | $156,000-$181,999 (i.e., $3,000-$3,499 a week) |  |
| **16** | $182,000-$207,999 (i.e., $3,500-$3,999 a week) |  |
| **17** | $208,000-$233,999 (i.e., $4,000-$4,499 a week) |  |
| **18** | $234,000-$259,999 (i.e., $4,500-$4,999 a week) |  |
| **19** | $260,000-$311,999 (i.e., $5,000-$5,999 a week) |  |
| **20** | $312,000-$415,999 (i.e., $6,000-$7,999 a week) |  |
| **21** | $416,000 or more (i.e., $8,000 or more a week) |  |
| **22** | Prefer not to answer |  |

# Section J: Quality of Life

**Below is a list of statements that other people with your illness have said are important. Please circle or mark one number per line to indicate your response as it applies to the past 7 days.**

PROGRAMMER: PLEASE REFER TO FACT-LEU SCORING GUIDELINES (VERSION 4) PLEASE REFERENCE QOL MEASURE AS: English (Universal); Copyright 1987,1997: 19 November 2007

|  | **PHYSICAL WELL-BEING** | **Not at all** | | **A little bit** | **Some-what** | **Quitea bit** | **Very much** |
| --- | --- | --- | --- | --- | --- | --- | --- |
|  |  |  |  |  |  |  |  |
| GP1 | I have a lack of energy | 0 | | 1 | 2 | 3 | 4 |
| GP2 | I have nausea | 0 | | 1 | 2 | 3 | 4 |
| GP3 | Because of my physical condition, I have trouble meeting the needs of my family | 0 | | 1 | 2 | 3 | 4 |
| GP4 | I have pain | 0 | | 1 | 2 | 3 | 4 |
| GP5 | I am bothered by side effects of treatment | 0 | | 1 | 2 | 3 | 4 |
| GP6 | I feel ill | 0 | | 1 | 2 | 3 | 4 |
| GP7 | I am forced to spend time in bed | 0 | | 1 | 2 | 3 | 4 |
|  | | | | | | | |
|  | **SOCIAL/FAMILY WELL-BEING** | **Not at all** | | **A little bit** | **Some-what** | **Quitea bit** | **Very much** |
|  |  |  |  |  |  |  |  |
| GS1 | I feel close to my friends | 0 | | 1 | 2 | 3 | 4 |
| GS2 | I get emotional support from my family | 0 | | 1 | 2 | 3 | 4 |
| GS3 | I get support from my friends | 0 | | 1 | 2 | 3 | 4 |
| GS4 | My family has accepted my illness | 0 | | 1 | 2 | 3 | 4 |
| GS5 | I am satisfied with family communication about my illness | 0 | | 1 | 2 | 3 | 4 |
| GS6 | I feel close to my partner (or the person who is my main support) | 0 | | 1 | 2 | 3 | 4 |
| Q1 | *Regardless of your current level of sexual activity, please answer the following question. If you prefer not to answer it, please mark this box and go to the next section.* | |  |  |  |  |  |
| GS7 | I am satisfied with my sex life | | 0 | 1 | 2 | 3 | 4 |

**Please circle or mark one number per line to indicate your response as it applies to the past 7 days.**

|  | **EMOTIONAL WELL-BEING** | **Not at all** | | **A little bit** | **Some-what** | **Quitea bit** | **Very much** |
| --- | --- | --- | --- | --- | --- | --- | --- |
|  |  |  |  |  |  |  |  |
| GE1 | I feel sad | | 0 | 1 | 2 | 3 | 4 |
| GE2 | I am satisfied with how I am coping with my illness | | 0 | 1 | 2 | 3 | 4 |
| GE3 | I am losing hope in the fight against my illness | | 0 | 1 | 2 | 3 | 4 |
| GE4 | I feel nervous | | 0 | 1 | 2 | 3 | 4 |
| GE5 | I worry about dying | | 0 | 1 | 2 | 3 | 4 |
| GE6 | I worry that my condition will get worse | | 0 | 1 | 2 | 3 | 4 |

|  | **FUNCTIONAL WELL-BEING** | **Not at all** | | **A little bit** | **Some-what** | **Quitea bit** | **Very much** |
| --- | --- | --- | --- | --- | --- | --- | --- |
|  |  |  |  |  |  |  |  |
| GF1 | I am able to work (include work at home) | | 0 | 1 | 2 | 3 | 4 |
| GF2 | My work (include work at home) is fulfilling | | 0 | 1 | 2 | 3 | 4 |
| GF3 | I am able to enjoy life | | 0 | 1 | 2 | 3 | 4 |
| GF4 | I have accepted my illness | | 0 | 1 | 2 | 3 | 4 |
| GF5 | I am sleeping well | | 0 | 1 | 2 | 3 | 4 |
| GF6 | I am enjoying the things I usually do for fun | | 0 | 1 | 2 | 3 | 4 |
| GF7 | I am content with the quality of my life right now | | 0 | 1 | 2 | 3 | 4 |

**Please circle or mark one number per line to indicate your response as it applies to the past 7 days.**

|  | **ADDITIONAL CONCERNS** | **Not at all** | **A little bit** | **Some-what** | **Quite**  **a bit** | **Very much** |
| --- | --- | --- | --- | --- | --- | --- |
|  |  |  |  |  |  |  |
| BRM3 | I am bothered by fevers (episodes of high body temperature) | 0 | 1 | 2 | 3 | 4 |
| P2 | I have certain parts of my body where I experience pain | 0 | 1 | 2 | 3 | 4 |
| BRM2 | I am bothered by the chills | 0 | 1 | 2 | 3 | 4 |
| ES3 | I have night sweats | 0 | 1 | 2 | 3 | 4 |
| LEU1 | I am bothered by lumps or swelling in certain parts of my body (e.g., neck, armpits, or groin) | 0 | 1 | 2 | 3 | 4 |
| TH1 | I bleed easily | 0 | 1 | 2 | 3 | 4 |
| TH2 | I bruise easily | 0 | 1 | 2 | 3 | 4 |
| HI12 | I feel weak all over | 0 | 1 | 2 | 3 | 4 |
| BMT6 | I get tired easily | 0 | 1 | 2 | 3 | 4 |
| C2 | I am losing weight | 0 | 1 | 2 | 3 | 4 |
| C6 | I have a good appetite | 0 | 1 | 2 | 3 | 4 |
| An7 | I am able to do my usual activities | 0 | 1 | 2 | 3 | 4 |
| N3 | I worry about getting infections | 0 | 1 | 2 | 3 | 4 |
| LEU5 | I feel uncertain about my future health | 0 | 1 | 2 | 3 | 4 |
| LEU6 | I worry that I might get new symptoms of my illness | 0 | 1 | 2 | 3 | 4 |
| BRM9 | I have emotional ups and downs | 0 | 1 | 2 | 3 | 4 |
| LEU7 | I feel isolated from others because of my illness or treatment | 0 | 1 | 2 | 3 | 4 |

# Section K: Feedback and Thank You

## Feedback

### K1: Feedback

#### Do you have any comments or suggestions for future surveys like this?

##### Please type in the box below

| OPEN TEXT BOX |
| --- |

### K2: Incentive [SINGLE] – hidden for panel

#### In appreciation of your time and contribution you will receive $70. Please select your preferred payment option below:

| **1** | E-gift card (this will be sent to the email address you provided earlier) | Use email address provided earlier: ^PIPE EMAIL^ |
| --- | --- | --- |
| **2** | Donate to advocacy (e.g. Lymphoma Australia) |  |

### K3: Summary of Findings [SINGLE] – hidden for panel

#### Would you like to receive a summary of findings upon the completion of this research?

##### Please choose which option you would prefer

| **1** | Yes (this will be sent to the email address you provided earlier) | Use email address provided earlier: ^PIPE EMAIL^ |
| --- | --- | --- |
| **2** | No |  |

### K4: Recontact consent [SINGLE] – hidden for panel

**Do you give permission to be recontacted to participate in future research? This will continue to help us in our understanding of what people experience due to living with CLL.**

##### Please select one answer

| **1** | Yes, I give permission for my email to be stored so I can be recontacted for future research |  |
| --- | --- | --- |
| **2** | No, I do not give permission for my email to be stored, so I can be recontacted for future research |  |

### K5: Thank you

#### Thank you for taking part in the survey. This research will help improve understanding about the attributes of treatment that are preferred by people living with CLL. The results will be used to guide future research and medicine funding activities. We also hope to publish the results so that others can learn from them. Remember to please see your doctor if you have any questions about your condition and its management. Please click on "Next" button to close the survey.

##### Please click "Next" to close the survey
